# Removing carbon catabolite repression in *Parageobacillus thermoglucosidasius* DSM 2542

**DOI:** 10.3389/fmicb.2022.985465

**Published:** 2022-10-20

**Authors:** Jinghui Liang, Richard van Kranenburg, Albert Bolhuis, David J. Leak

**Affiliations:** ^1^Department of Biology and Biochemistry, University of Bath, Bath, United Kingdom; ^2^Centre for Sustainable and Circular Technologies (CSCT), University of Bath, Bath, United Kingdom; ^3^Laboratory of Microbiology, Wageningen University, Wageningen, Netherlands; ^4^Corbion, Gorinchem, Netherlands; ^5^Department of Pharmacy and Pharmacology, Centre for Therapeutic Innovation, University of Bath, Bath, United Kingdom

**Keywords:** carbon catabolite repression, *Parageobacillus thermoglucosidasius*, 2-deoxyglucose resistance, adaptive evolution, mixed-sugar fermentation

## Abstract

*Parageobacillus thermoglucosidasius* is a thermophilic bacterium of interest for lignocellulosic biomass fermentation. However, carbon catabolite repression (CCR) hinders co-utilization of pentoses and hexoses in the biomass substrate. Hence, to optimize the fermentation process, it is critical to remove CCR in the fermentation strains with minimal fitness cost. In this study, we investigated whether CCR could be removed from *P. thermoglucosidasius* DSM 2542 by mutating the Ser46 regulatory sites on HPr and Crh to a non-reactive alanine residue. It was found that neither the *ptsH1* (HPr-S46A) nor the *crh1* (Crh-S46A) mutation individually eliminated CCR in *P. thermoglucosidasius* DSM 2542. However, it was not possible to generate a *ptsH1 crh1* double mutant. While the Crh-S46A mutation had no obvious fitness effect in DSM 2542, the *ptsH1* mutation had a negative impact on cell growth and sugar utilization under fermentative conditions. Under these conditions, the *ptsH1* mutation was associated with the production of a brown pigment, believed to arise from methylglyoxal production, which is harmful to cells. Subsequently, a less directed adaptive evolution approach was employed, in which DSM 2542 was grown in a mixture of 2-deoxy-D-glucose(2-DG) and xylose. This successfully removed CCR from *P. thermoglucosidasius* DSM 2542. Two selection strategies were applied to optimize the phenotypes of evolved strains. Genome sequencing identified key mutations affecting the PTS components PtsI and PtsG, the ribose operon repressor RbsR and adenine phosphoribosyltransferase APRT. Genetic complementation and bioinformatics analysis revealed that the presence of wild type *rbsR* and *apt* inhibited xylose uptake or utilization, while *ptsI* and *ptsG* might play a role in the regulation of CCR in *P. thermoglucosidasius* DSM 2542.

## 1. Introduction

Producing energy and chemicals from fossil fuels is not sustainable, and therefore several alternative approaches have been considered, among which microbial fermentation of renewable feedstocks has gained growing interest (Wee et al., 2006; Wang et al., 2010). Renewable lignocellulosic feedstocks such as purpose grown crops from non-arable land or waste lignocellulosic biomass are particularly attractive feedstocks (Vishnu et al., 2002; Wee et al., 2006) as it is abundant and readily available in many parts of the world (Oh et al., 2005; Banerjee et al., 2010). Lignocellulose derived from plants mainly consists of cellulose [40–60% w/w(dry)], hemicellulose (20-40%) and lignin (10-20%). After pre-treatment and hydrolysis, a mixture of C6 and C5 sugars is released, including glucose, galactose, mannose, xylose and arabinose. These sugars can be fermented using methods similar to those of first-generation feedstocks; however, some microbes are not able to grow on C5 sugars (Kang et al., 2014). Hence, the ability to utilize both C6 and C5 sugars is an important trait when selecting strains for lignocellulosic biomass fermentation (Banerjee et al., 2010). To date, the most common microbial process organisms are mesophilic, such as *Escherichia coli, Lactobacillus*, and *Saccharomyces* (Banerjee et al., 2010; Mattanovich et al., 2014). However, it has been suggested that certain thermophilic bacteria are more suitable for lignocellulosic biomass fermentation, as a fermentation process operating at 55–60°C is more suited for simultaneous saccharification and fermentation (SSF), improves the energy efficiency of this exothermic process and reduces contamination risks (Bacon et al., 2017). High temperatures also facilitate the removal of volatile products (Hills, 2015). Many thermophilic microbial species have been investigated, including *Geobacillus* and *Parageobacillus* spp. These are of industrial interest because, not only can they ferment the most abundant hexose and pentose monomers in lignocellulose, but also cellobiose and complex xylo-oligosaccharides present in pretreated lignocellulosic biomass (Reeve et al., 2016; Sheng et al., 2016). For example, *P. thermoglucosidasius* has been engineered for bio-ethanol, terpenes and (S)-lactic acid production (Atkinson et al., 2013; Van Kranenburg et al., 2019; Styles et al., 2021).

Carbon catabolite control enables the preferential utilization of the most energy-efficient carbon source, so that microbes can achieve a higher growth yield but retain the metabolic flexibility to survive in a nutritionally fluctuating environment (Martin-Verstraete et al., 1999; Görke and Stülke, 2008). For optimal metabolic efficiency in a competitive environment, metabolism of the preferred carbon source, typically glucose, constrains the expression of genes responsible for catabolism of those less-favored carbon sources; this regulatory phenomenon is called carbon catabolite repression (CCR) (Görke and Stülke, 2008; Vinuselvi et al., 2012). It is also known as the glucose effect, because the existence of glucose often represses the consumption of other carbon sources, although this is not universally the case (Shaw et al., 2008; VanFossen et al., 2009). In fermentation of plant-derived feedstocks, where carbon sources are naturally combined in different proportions, sequential consumption of sugars resulting from CCR leads to extended fermentation times and differences in metabolism of different sugars can make it challenging to control the fermentations using engineered strains efficiently (Kim et al., 2010). Also, where the less favored sugar is metabolized too slowly, unused substrate might complicate the downstream processing and affect the purity of final products (Vinuselvi et al., 2012). Strain development for fermentation of lignocellulosic biomass has usually been focused on increasing product yields. However, the simultaneous consumption of all sugars in the substrate is important for process design and can benefit overall productivity (Kim et al., 2010). Hence, elimination of CCR in microbial cell factories is desirable (Liu et al., 2014). While there are reports of removing CCR in *Bacillus* spp. (Kraus et al., 1994; Martin-Verstraete et al., 1995; Sun and Altenbuchner, 2010; Nathan and Nair, 2013), it is not evident that these strategies apply to *Parageobacillus* spp.

In Firmicutes, a histidine-containing phosphocarrier protein (HPr) and an HPr-like protein Crh are key players in CcpA (catabolic control protein A)-dependent CCR. When HPr is phosphorylated on a catalytic histidine residue at position 15, this phosphate can subsequently be transferred to the preferred sugar as it is imported across the cell membrane *via* a phosphotransferase system (PTS) (Liang et al., 2021). According to the accepted model, an elevated concentration of glycolytic intermediates, glucose-6-phosphate (G-6-P) and fructose-1,6-biphosphate (FBP), stimulates the regulatory phosphorylation of HPr at a serine residue at position 46 by an ATP-dependent HPr kinase (Deutscher et al., 1995; Gunnewijk and Poolman, 2000; Görke and Stülke, 2008; Sun and Altenbuchner, 2010). Crh displays a high sequence identity and high structural similarity to HPr. However, Crh does not have a catalytic His15 residue, which is replaced by a glutamine residue in *P. thermoglucosidasius*, though it can be phosphorylated by the HPr kinase at Ser46 and thereby invokes CCR (Schumacher et al., 2007; Sun and Altenbuchner, 2010). The HPr (Ser-P) or Crh (Ser-P) binds to the catabolic control protein A (CcpA) to form a CcpA-HPr (Ser46-P) or CcpA-Crh (Ser46-P) complex, which binds to an operator sequence (catabolite responsive element [*cre*]) located 5' to, or within catabolic genes and results in the down-regulation of target operons (Lorca et al., 2005; Schumacher et al., 2006). Based on this mechanism, several strategies have been investigated to eliminate CCR in *Bacillus* spp. It has been demonstrated that when the Ser46 residue on HPr is replaced by an alanine residue (*ptsH1* mutation), the HPr can no longer be phosphorylated at this position (Hueck and Hillen, 1995; Fujita, 2009). In *B. subtilis*, several catabolic genes were partially or completely relieved from CCR by the HPr-S46A mutation (Deutscher et al., 1994; Dahl and Hillen, 1995; Reizer et al., 1996). Gene disruption or an alanine substitution of Crh-Ser46 (*crh1* mutation) alone did not mitigate CCR, but when such mutation was introduced into a *ptsH1* background, the residual CCR effect observed in some operons (e.g., *xyn, lev*, and *iol*) completely disappeared, suggesting that Crh has a back-up function to HPr (Galinier et al., 1999; Martin-Verstraete et al., 1999; Puri-Taneja et al., 2006; Repizo et al., 2006). It has been suggested that the Crh in *B. subtilis* is also responsible for some regulatory functions which are not shared by HPr.

To date, various strategies have been designed to obtain mutant strains which are resistant to CCR. In *B. subtilis*, it has been reported that several genes could be relieved from CCR when the Ser46 residue was replaced by an Ala on both HPr and Crh. *P. thermoglucosidasius* is a member of the *Bacillaceae* family, and its genome sequence shows that it has the potential to produce all the key proteins involved in CCR. Hence, in principle, the mechanism of CCR should be similar in these two organisms. However, as illustrated in this work, a *ptsH1* mutant of *P. thermoglucosidasius* DSM2542 showed impaired growth under fermentative conditions, so we resorted to a classical non-targeted approach based on resistance to the catabolite repressing substrate analog, 2-deoxyglucose (2-DG).

2-DG is a non-metabolizable synthetic glucose analog where the 2-hydroxyl group has been replaced by hydrogen (Horton et al., 1973; Zhu et al., 2015). Due to its similarity to glucose, 2-DG can be recognized by the PTS or other transport systems for transport into cells (Zhu et al., 2015). After phosphorylation at the available 6 position, the resulting 2-deoxy-D-glucose-6-phosphate (2-DG-6P) would not be further metabolized due to the lack of a hydroxyl group at the 2 position (Xi et al., 2014). Hence, cells cannot grow on 2-DG but the accumulation of 2-DG-6P could act as a catabolite repression signal, meaning that the cells would also not be able to grow on a less favored carbon source in the presence of 2-DG. Several studies have demonstrated that this concept is borne out in practice, with 2-DG inducing CCR in *S. cerevisiae, E. coli*, and *Bacillus* spp., repressing the utilization of raffinose, fructose, and soluble starch, respectively (Zimmermann and Scheel, 1977; Do et al., 1993; Kornberg and Lambourne, 1994). Use of a non-metabolizable analog such as 2-DG has previously been shown to provide a platform for the isolation of CCR-resistant strains which are able to grow on the less favored substrate in the presence of the analog (Do et al., 1993). In the present work, an adaptive evolution approach was employed to remove CCR from *P. thermoglucosidasius* DSM 2542. According to previous studies, CCR in *P. thermoglucosidasius* DSM 2542 is not as stringent as that commonly seen in other microbes, because it can still metabolize xylose slowly in the presence of glucose (Liang et al., 2021). Nevertheless, this still provides a platform to isolate CCR resistant mutants based on faster growth on xylose than the parent strain, in the presence of 2-DG. Based on this principle, two evolution experiments are described, which successfully generated CCR-resistant *P. thermoglucosidasius* DSM 2542 mutants. Whole genome sequencing of these strains and genetic complementation analysis revealed the genes which contribute to the phenotypes observed.

## 2. Materials and methods

### 2.1. Bacterial strains, plasmids, primers, standard reagents, and bacterial growth medium used in this study

The following bacterial strains (Table 1), plasmids (Table 2), and primers (Table 3) were used in this study. All reagents were purchased from Sigma Aldrich (Dorset, UK) or Fisher Scientific (Loughborough, UK). Cell culture media were prepared as described previously (Liang et al., 2021). A final kanamycin concentration of 50 μg/mL was used for *E. coli* and 12.5 μg/mL was used for *P. thermoglucosidasius*.

**Table 1:** Bacterial strains used in this study.

**Strain**	**Description**	**Source**
*P. thermoglucosidasius* DSM 2542	Wild type strain	DSMZ, Germany
*P. thermoglucosidasius* DSM 2542 *ptsH1*	HPr-S46A variant of *P. thermoglucosidasius* DSM 2542	This study
*P. thermoglucosidasius* DSM 2542 *crh1*	Crh-S46A variant of *P. thermoglucosidasius* DSM 2542	This study
*P. thermoglucosidasius* TM444	Variant of *P. thermoglucosidasius* NCIMB 11955 (*ldh*A, *pfl*B, *P*–*ldh* (NCA1503)/*pdh*_*up*_, spo0A)	TMO Renewables, collection held at University of Bath
*P. thermoglucosidasius* TM444 Δ*ptsH*	Variant of *P. thermoglucosidasius* NCIMB 11955 (*ldh*A, *pfl*B, *P*–*ldh* (NCA1503)/*pdh*_*up*_, *spo0*A, *pts*H)	TMO Renewables, collection held at University of Bath
2DG-ADE1 (a,b,c,d,e,f)	Evolved strains of *P. thermoglucosidasius* DSM 2542 from the first 2-DG evolution	This study
2DG-ADE2 (a,b,c,d,e)	Evolved strains of *P. thermoglucosidasius* DSM 2542 from the second 2-DG evolution	This study
*E. coli* DH5α	Cloning strain, F-, *φ80lacZΔM15 Δ(lacZYA-argF)* U169 *recA1 endA1 hsdR17 (rK− mK+) phoA supE44 λ- thi-1 gyrA96 relA1*	David Leak Lab, University of Bath
*E. coli* S17-1	Tp^*R*^ Sm^*R*^ recA, thi, *pro*, hsdR-M+RP4:2-Tc:Mu:Km Tn7 λpir	David Leak Lab, University of Bath Simon et al., 1983

**Table 2:** Plasmids used in this study.

**Plasmid**	**Description**	**References**
pCR*^*TM*^*-Blunt	Vector from Zero Blunt PCR cloning kit	Invitrogen, Dublin, Ireland
pCR*^*TM*^*-Blunt-ptsH	pCR^*TM*^-Blunt + DSM 2542 *ptsH*	This study
pCR^*TM*^-Blunt-crh	pCR^*TM*^-Blunt + DSM 2542 *crh*	This study
pCR^*TM*^-Blunt-ptsH1	pCR^*TM*^-Blunt + *ptsH*1 (HPr-S46A point mutation)	This study
pCR^*TM*^-Blunt-crh1	pCR^*TM*^-Blunt + *crh*1 (Crh-S46A point mutation)	This study
pG2K-*oriT*-*bgl*-*sfgfp*-GG	Cloning vector modified from pG2K-*oriT*-*bgl*-*sfgfp*, containing a km^*R*^ selection marker and *Bsa*I (Golden Gate) cloning sites	Bacon et al., 2017, Ortenzi, unpublished
pJL-*ptsH1*	pG2K-*oriT*-*bgl*-*sfgfp*-GG containing *ptsH*1 and flanking regions of *ptsH* from *P. thermoglucosidasius* DSM 2542	This study
pJL-*crh1*	pG2K-*oriT*-*bgl*-*sfgfp*-GG containing *crh*1 and flanking regions of *crh* from *P. thermoglucosidasius* DSM 2542	This study
pJL-*ptsG^+^*	pG2K-*oriT*-*bgl*-*sfgfp*-GG containing the wild type *ptsG* from *P. thermoglucosidasius* DSM 2542	This study
pJL-*rbsR^+^*	pG2K-*oriT*-*bgl*-*sfgfp*-GG containing the wild type *rbsR* from *P. thermoglucosidasius* DSM 2542	This study
pJL-*apt^+^*	pG2K-*oriT*-*bgl*-*sfgfp*-GG containing the wild type *apt* from *P. thermoglucosidasius* DSM 2542	This study

**Table 3:** Primers used in this study.

**Oligonucleotide name**	**Sequence^*a*^**	**Purpose**
1Fw	aggtctccacagggccgactgatggaacgg	Amplify upstream-*ptsH*
1Rv	aggtctcgctagagagataagacttgggttggaaatcg	downstream-*ptsH*
2Fw	cggtaaacttaaaa**gcg**atcatgggtgttatgtcattaggaattcc	*ptsH1* site-directed
2Rv	cacccatgat**cgc**ttttaagtttaccgtttttccattatactcc	mutagenesis
3Fw	aggtctcgctagattatcattcttggcagtcc	Amplify upstream-*crh*
3Rv	aggtctccacatttcttaatgctaaaacgctg	downstream-*crh*
4Fw	gaatgcaaaa**gct**attatggggcttatgagcctcgcaatc	*crh1* site-directed
4Rv	cccataat**agc**ttttgcattcactcttttcccgtctttttcc	mutagenesis
5Fw	ccattttcggtccaaagtcc	Amplify from upstream and downstream of
5Rv	aatgacgttaccgtccaatcc	to check *ptsH*1 integration on chromosome
6Fw	ttgtgcgaatcgtcgacg	Amplify from upstream and downstream of
6Rv	atgtcttatgccggcagc	to check *crh*1 integration on chromosome
7Fw	tgggattcaggagtggacag	Amplify from upstream and downstream of *Bsa*I cloning
7Rv	cggctcgtatgttgtgtgg	sites to check the presence of plasmid and insert
8Fw	aGGTCTCCACAGtgtctctctttacgtgttac	upstream-*ptsG*
8Rv	aGGTCTCGCTAGcaattgacaaaagtaaatttacaag	downstream-*ptsG*
9Fw	aGGTCTCCACAGcaccggtttcgtcattttc	upstream-*rbsR*
9Rv	aGGTCTCGCTAGcgcaatatgtatgttctcttg	downstream-*rbsR*
10Fw	aGGTCTCCACAGtttgtttagatggccgct	upstream-*apt*
10Rv	aGGTCTCGCTAGggcaaaacaaccgtaataag	downstream-*apt*
M13Fw	gtaaaacgacggcca	Check the presence of pCR^*TM*^-Blunt plasmid and insert
M13Rv	caggaaacagctatgacc	

^*a*^Bold letters highlight the point mutation site.

### 2.2. Standard reagents and bacterial growth medium

All reagents were purchased from Sigma Aldrich (Dorset, UK) or Fisher Scientific (Loughborough, UK). Cell culture media were prepared as described previously (Liang et al., 2021). A final kanamycin concentration of 50 μg/mL was used for *E. coli* and 12.5 μg/mL was used for *P. thermoglucosidasius*.

### 2.3. Genomic DNA preparation and site-directed mutagenesis

Genomic DNA from *P. thermoglucosidasius* DSM 2542 was extracted with a Wizard Genomic DNA Purification Kit (Promega, Hampshire, UK) according to manufacturer's instructions. The DNA concentration was quantified using a NanoVue Plus spectrophotometer (Biochrom, Cambridge, UK). The *ptsH* and *crh* genes along with their flanking regions of about 400 bp were amplified from the genomic DNA with Phusion high-fidelity polymerase (Fisher Scientific, Loughborough, UK) following the manufacturer's protocol using primers 1Fw and 1Rv for *ptsH*, and primers 3Fw and 3Rv for *crh*, followed by cloning into a pCR^*TM*^-Blunt vector (Zero Blunt PCR cloning kit, Invitrogen, Dublin, Ireland). Chemically competent *E. coli* DH5α cells were prepared and transformed with the ligation mixture by heat shock as described previously (Chang et al., 2017). The transformants were screened for the presence of the plasmid insert by colony PCR with MyTaq 2x Red Mix (Bioline, London, UK) using primers M13Fw and M13Rv. Plasmids were then extracted from overnight liquid cultures of selected transformants with a QIAprep Spin Miniprep Kit (Qiagen, Manchester, UK) and the presence of the correct insert confirmed by sequencing (GATC services, Eurofins Genomics, Cologne, Germany). After that, primers 2Fw and 2Rv or 4Fw and 4Rv encoding the Ser46 to alanine mutation were used to amplify the pCR^*TM*^-Blunt-*ptsH* and pCR^*TM*^-Blunt-*crh* plasmid, respectively, with a modified Phusion high-fidelity polymerase protocol (98°C for denaturation, 60°C for annealing, 1 min/kb for extension at 72°C, 12 cycles). Twenty nanograms of the template plasmid and 50 ng of each primer were used in each reaction. After PCR, the reaction mix was incubated with 0.5 μL FastDigest *Dpn*I (Thermo Scientific, Paisley, UK) at 37°C for 60 min, to digest the plasmid template. Next, chemically competent *E. coli* DH5α cells were transformed with the amplified plasmid mixture. Plasmid DNA was extracted from the transformants and sent for sequencing to confirm the generation of the desired pCR^*TM*^-Blunt-*ptsH1* and pCR^*TM*^-Blunt-*crh1* constructs.

### 2.4. Golden gate assembly

The *ptsH1* and *crh1* sequences along with their flanking regions were digested from the pCR^*TM*^-Blunt-*ptsH1* and pCR^*TM*^-Blunt-*crh1* construct, respectively, with FastDigest *Bsa*I (Thermo Scientific, Paisley, UK), and purified by gel electrophoresis. The purified fragments were cloned into the vector pG2K-*oriT*-*bgl*-*sfgfp*-GG (Ortenzi, unpublished), respectively, *via* Golden Gate Assembly (Engler et al., 2009; Reeve et al., 2016). Each 15 μL reaction mixture contains 300 ng of the vector backbone and equimolar concentration of insert, 1.5 μL FastDigest Buffer, 1 μL FastDigest *Bsa*I, 2 μL NEB T4 ligase (400,000 units/mL), 1.5 μL T4 ligase buffer and nuclease-free ddH_2_O. The reaction was carried out in a thermocycler to repeat the digestion (37°C, 3 min) and ligation (16°C, 4 min) for 50 times, followed by 50°C for 5 min and 80°C for 5 min, then stored at 8°C. Chemically competent *E. coli* DH5α cells were transformed with each reaction mix and transformants selected on LB agar plates containing 50 μg/mL kanamycin. Successful integration of the mutant genes, forming pJL-*ptsH1* or pJL-*crh1* was confirmed by sequencing (GATC services, Eurofins Genomics, Cologne, Germany).

### 2.5. Conjugation and chromosomal integration

Chemically competent *E. coli* S17-1 cells were prepared and transformed with the pJL-*ptsH1* and pJL-*crh1* plasmids by heat-shock as described previously (Chang et al., 2017). Transformants were selected on LB agar plates containing 50 μg/mL kanamycin. Successful transformation was confirmed by colony PCR with primer 7Fw and 7Rv. After conjugation into DSM 2542 as described previously (Macklyne, 2017; Liang et al., 2021), the presence of the insert in the plasmids pJL-*ptsH1* and pJL-*crh1* was confirmed by colony PCR with primer 7Fw and 7Rv. The protocol for selection of strains in which a gene has integrated into the chromosome *via* homologous recombination relies on a temperature sensitive replicon encoded by *repB* which is inactive above 65°C (Reeve et al., 2016). Firstly, the colonies that had been grown at 52°C were inoculated into 10 mL TGP medium (12.5 μg/mL kanamycin) and grown for 6–8 h at 68°C, and sub-cultured twice to ensure loss of the native plasmid but retention of the Km^*R*^ (kanamycin resistance marker) through homologous recombination *via* one of the flanking regions. The primary integration was confirmed by colony PCR of colonies grown on TGP plates containing 12.5 μg/mL kanamycin. Then, colonies with successful primary integration were inoculated into 10 mL TGP medium without antibiotics and sub-cultured every 10–14 h at 52°C to allow for the second homologous recombination which removes the integrated plasmid, leaving either the mutant or native gene at the original site of integration. One hundred microliters of the 6th sub-culture was serially diluted (10^−1^, 10^−3^, 10^−5^, 10^−7^) and plated onto TGP agar plates with no antibiotics. After growth on TGP plates overnight at 52°C, colonies were then replica plated onto TGP agar plates with and without kanamycin and incubated at 52°C overnight. Kanamycin sensitive colonies were screened by PCR, using primers 5Fw and 5Rv for *ptsH*, and 6Fw and 6Rv for *crh*, respectively. The *ptsH1* mutation removed a *Bsu*15I restriction site within *ptsH*, while the *crh1* mutation introduced an *Alu*I restriction site in the *crh* (Supplementary Figure 2A). Hence, digestion of the PCR products by FastDigest *Bsu*15I or *Alu*I (Thermo Scientific, Paisley, UK) was used to confirm the presence of the desired *ptsH1* or *crh1* mutation.

### 2.6. Construction and conjugation of vectors for genetic complementation

The wild type *ptsG, rbsR*, and *apt* along with their native promoters were individually amplified from *P. thermoglucosidasius* DSM 2542 genomic DNA using primers listed in Table 3 with Phusion high-fidelity polymerase (Fisher Scientific, Loughborough, UK) following the manufacturer's protocol. Each of the gene fragments, purified by gel electrophoresis, were cloned into a pair of *Bsa*I sites on the plasmid pG2K-*oriT*-*bgl*-*sfgfp*-GG (Ortenzi, unpublished) *via* Golden Gate Assembly (Engler et al., 2009; Reeve et al., 2016). Chemically competent *E. coli* DH5α cells were prepared and transformed with the reaction mixes, respectively, by heat shock as described previously (Chang et al., 2017). The presence of the requisite pJL-*ptsG*^+^, pJL-*apt*^+^, and pJL-*rbsR*^+^ constructs was confirmed by colony PCR of transformants grown on LB agar plates containing 50 μg/mL kanamycin with primers 7Fw and 7Rv, then the amplified product was authenticated by sequencing (GATC services, Eurofins Genomics, Cologne, Germany). Then, 2DG-ADE2b was transformed with pJL-*ptsG*^+^, pJL-*apt*^+^, and pJL-*rbsR*^+^, respectively, *via* conjugation as described previously (Liang et al., 2021). The continued presence of the gene inserts in the 2DG-ADE2b transformants was confirmed by colony PCR.

### 2.7. Tube fermentation

Tube fermentation was carried out in 15 mL sterile conical centrifuge tubes containing 10 mL ASM medium and 0.2 g solid MgCO_3_ (sterilized by autoclave) to stabilize the pH (Millard et al., 1996). *P. thermoglucosidasius* strains were revived from glycerol stocks and grown on 15 mL TGP or 2TY plates overnight at 60°C. The next morning, a loopful of cells was inoculated into 15 mL TGP or 2TY liquid medium and incubated for 4–5 h (60°C, 250 rpm) until the OD_600_ optical density measured at a wavelength of 600 reached 2.5~3.0. 1 mL of the cell culture was inoculated into each tube, and the cultures were incubated at 60°C in a shaking incubator at 250 rpm. The relatively small head space in the tubes allowed for the transition from fully aerobic to fermentative conditions as oxygen transfer became limited. Experiments were done in triplicate and samples taken every 24 h in sacrifice tubes, centrifuged and the clarified supernatant analyzed by HPLC as described previously (Hills, 2015; Liang et al., 2021).

### 2.8. GC-MS identification of methylgloxal in fermentation broth

The identification of methylglyoxal was based on the formation of 2-methylquinoxaline after reaction with 1,2-diaminobenzene as described previously (OIV-MA-AS315-21, 2010). After tube fermentation in ASM medium [1% (w/v) glucose and 1% (w/v) xylose] for 48 h, cell cultures of *P. thermoglucosidasius* DSM 2542 and DSM 2542 *ptsH1* were filtered (PhenexNY 15mm Syringe Filters 0.2 μm, Phenomenex, Torrance, USA). 10 mL of each clarified filtrate was brought to pH 8.0 with 1M NaOH, followed by derivatization with 5 mg 1,2-diaminobenzene for 3 h at 60°C. Then the reaction medium was brought to pH 2.0 using 2 M H_2_SO_4_, and the quinoxaline derivative was extracted twice with 1 mL dichloromethane. The solvent phase was dried on 0.2 g of anhydrous sodium sulfate prior to GC-MS analysis.

The reaction product was analyzed on an Agilent 8890 gas chromatography system coupled with 5977B MSD. Split injections (1 μL) were performed, with a split ratio of 30:1 (split flow 30 mL/min), using with a single taper, ultra-inert wool inlet liner (Agilent 5190-2293). The inlet was heated to 260°C with 3 mL/min septum purge flow. An Agilent HP-5MS (30 m, 0.25 mm, 0.25 μm) column was used for separation, with He (BOC, N5.5) carrier gas at a constant flow of 1.0 mL/min. The oven was held at 70°C for 1 min, thereafter ramped at 10°C/min to 200°C, then 20°C/min to 220°C holding for 2 min. The MS transfer line was set to 280°C with the source at 230°C and quad at 150°C. MSD detection was performed (after solvent delay of 6 min) using full scan mode from 40 to 400 m/z with 1562 μs scan speed, and a gain factor of 15. The ion m/z = 144 was used for quantification and the ion m/z = 117 as a qualifier of the derivatized methylglyoxal.

### 2.9. Methylglyoxal and ASM medium reaction assay

Fifty milliliters of *P. thermoglucosidasius* DSM 2542 culture in ASM medium (1% glucose and 1% xylose) at an OD_600_ of 0.2 was prepared in a 250 mL baffled Erlenmeyer flask. The cell culture was divided into 5 equal portions and transferred into 15 mL conical centrifuge tubes. Then, methylglyoxal was added to all except for one as a negative control. The final concentration (v/v) of methylglyoxal was 0, 0.1, 0.2, 0.3, and 0.4%, respectively. Cell cultures were incubated at 60°C. Color change of the cultures was documented routinely.

### 2.10. 2-DG repression assays

*P. thermoglucosidasius* DSM 2542 cells grown to exponential phase in ASM medium supplemented with 1% (w/v) xylose were inoculated into 50 mL sterile conical centrifuge tubes (Greiner, Stonehouse, UK) containing 20 mL ASM medium with 1% (w/v) xylose and different concentrations of 2-DG. The cultures were grown at 60°C in a shaking incubator (Innova44, New Brunswick, UK). Samples were removed at regular intervals and the OD_600_ was recorded. The inhibitory effect of 2-DG to *P. thermoglucosidasius* DSM 2542 during growth on xylose was determined by comparison of growth rates.

### 2.11. First adaptive evolution in ASM medium with 1% xylose and 0.5% 2-DG

Adaptive evolution of *P. thermoglucosidasius* DSM 2542 was performed in 50 mL sterile conical centrifuge tubes containing 20 mL ASM medium supplemented with 1% xylose and 0.5% 2-DG. Starting with an OD_600_ of 0.1, the cell culture was incubated in a shaking incubator (60°C, 250 rpm) and 1 mL of the culture was inoculated into a fresh tube every 24 h. These serial subcultures were analyzed every 7 days using tube fermentation to see if the cells were catabolite de-repressed. After 14 subcultures, single colonies were isolated on a 2TY agar plate from the catabolite de-repressed population, and their phenotypes were evaluated in tube fermentations. Genome sequencing of the parent strain and the evolved mutant strains was performed using the standard whole genome sequencing service provided by MicrobesNG (Birmingham, UK) carried out in Illumina sequencers (HiSeq/NovaSeq) using a 250 bp paired end protocol, followed by trimmed-reads and assembly using the existing sequence of DSM 2542 as a scaffold. The parent strain was also re-sequenced as a control. Variant calling was performed using VarScan. The sequences of the mutant strains were compared with the parent strain for single nucleotide polymorphisms (SNPs).

### 2.12. Second adaptive evolution with one selection on glucose

Cells were prepared and sub-cultured as described above. The only difference was that after 7 sub-cultures, 1 mL of cell culture was inoculated into ASM medium supplemented with 1% (w/v) glucose and grown for 10 h (60°C, 250 rpm). Then,1 mL of this cell culture was inoculated into ASM medium supplemented with 1% (w/v) xylose and 0.5% (v/v) 2-DG and sub-culturing in this medium every 24 h continued. Single colonies were isolated from the catabolite de-repressed population after 14 subcultures, and their phenotypes were evaluated in tube fermentations. Mutations in evolved strains were identified *via* genome sequencing and comparison with the parent strain as described above (MicrobesNG, Birmingham, UK).

## 3. Results

### 3.1. HPr-S46A and Crh-S46A site-directed mutagenesis in *P. thermoglucosidasius* DSM 2542

The protocol for chromosomal integration relies on a temperature sensitive replicon encoded by *repB* which is inactive above 65°C (Reeve et al., 2016). After the two homologous recombination events as shown in Supplementary Figure 1, kanamycin sensitive colonies were screened by PCR. Primers 5Fw and 5Rv were used to amplify the *ptsH* or *ptsH1* along with their flanking regions (about 1.7 kb in total), and primers 6Fw and 6Rv were used to amplify the *crh* or *crh1* along with their flanking regions (about 2 kb in total). *ptsH1* and *crh1* point mutation in *P. thermoglucosidasius* DSM 2542 was diagnosed by PCR and enzymatic digestion (Supplementary Figure 2). In wild type DSM 2542 background, 5 out of 16 screened colonies contained the desired *ptsH1* mutation, and 7 out of 15 screened colonies contained the desired *crh1* mutation, respectively. However, although several attempts have been made to create an *ptsH1* and *crh1* double mutant, none was successful. In attempts to mutate either *ptsH* in DSM 2542 *crh1*, or *crh* in DSM 2542 *ptsH1*, none of approximate 100 tested colonies contained the desired double mutation. During deletion of the second gene, the first crossover occurred but the second crossover did not result in the desired deletion.

### 3.2. Tube fermentations of *P. thermoglucosidasius* strains in mixed-sugar substrate

**Figure 1:**
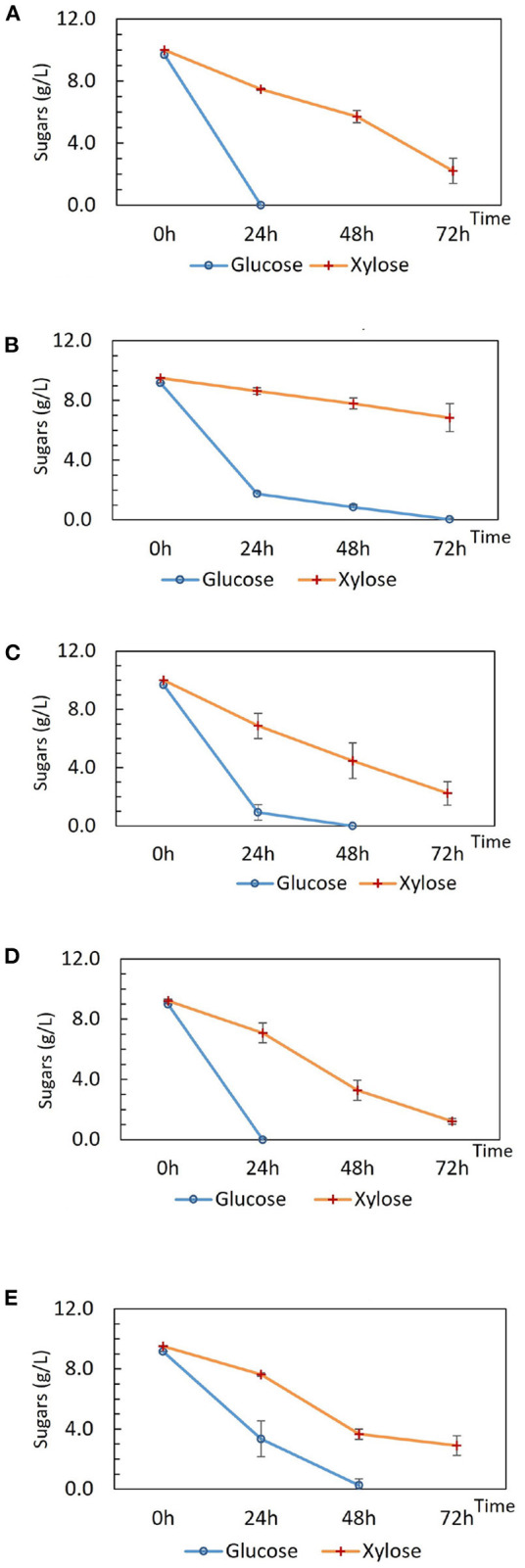
Sugar consumption of **(A)** wild type *P. thermoglucosidasius* DSM 2542, **(B)** *P. thermoglucosidasius* DSM 2542 *pstH1*, **(C)** P. thermoglucosidasius DSM 2542 *crh1*, **(D)** *P. thermoglucosidasius* TM444, and **(E)** *P. thermoglucosidasius* TM444 △*ptsH* during tube fermentation in sterile 15 mL centrifuge tubes. Cells were cultured in 10 mL ASM medium supplemented with 1% (w/v) glucose and 1% (w/v) xylose. To stabilize the pH during the fermentation, 0.2 g solid MgCO_3_ was added to the tubes prior to inoculation. OD_600_ readings were not possible because of the turbidity of the media caused by the MgCO_3_. Error bars are ± standard deviation of three replicates taken at the same time point.

Tube fermentations were set up for preliminary characterization of DSM 2542 *crh1* and DSM 2542 *ptsH1*. DSM 2542 *ptsH1* and DSM 2542 *crh1* prioritized the utilization of glucose in the first 24 h as observed in the wild type (Figures 1A–C), indicating that the S46A mutation in Crh or HPr alone, did not alleviate CCR during fermentative metabolism. Intriguingly, the extent of sugar consumption of these two mutant strains differed after 24 h. DSM 2542 *crh1* consumed all of the glucose within 48 h and about 80% of the xylose by 72 h. In contrast, though DSM 2542 *ptsH1* used most of the glucose in the first 24 h, its glucose consumption slowed down afterwards, with a dark-brown color developing in the media (which was not seen in DSM 2542 or DSM 2542 *crh1*). It only finished using the glucose between 48 and 72 h, with only 16% of the xylose consumed by 72 h, suggesting that xylose utilization was still being repressed when glucose was present. The slow glucose utilization after 24 h implied the *ptsH1* mutation might have resulted in a fitness cost which became apparent after about 24 h of growth and affected metabolism of both sugars.

TM444 is a *P. thermoglucosidasius* NCIMB 11955 variant with deletions to the genes encoding lactate dehydrogenase (Ldh), pyruvate formate lyase (PflB), stage 0 sporulation protein A (Spo0A), and up-regulation of the pyruvate dehydrogenase complex (Pdh) (Hills, 2015). TM444 △*ptsH* is a TM444 variant with a *ptsH* partial deletion (Bacon, unpublished). DSM 2542 and NCIMB 11955 are the same strain although a small number of single nucleotide polymorphisms (SNPs) between the strains have been recorded (Sheng et al., 2016). It was reported that TM444△*ptsH* was able to utilize pentose and hexose sugars simultaneously, yet it experienced slow growth and slow sugar consumption when compared with wild type NCIMB 11955 under fermentative conditions but not in aerobic conditions (Marriot, unpublished). Here, to investigate the impact of △*ptsH* on sugar consumption and medium color during tube fermentation, TM444 △*ptsH* was compared with TM444 in ASM medium supplemented with glucose and xylose (Figures 1D,E) with a focus on glucose consumption and medium color. While TM444 was similar to DSM2542 with respect to CCR, TM444 △*ptsH* appeared to metabolize glucose and xylose simultaneously, but primarily because glucose was consumed more slowly than in TM444. Xylose was consumed at a similar rate to the wild-type and neither culture produced the dark brown color seen with DSM 2542 *ptsH1*. Thus, the phenotype of DSM 2542 *ptsH1* seems to be specific to a catalytically functional but signaling uncoupled form of PtsH. This metabolized glucose as rapidly as DSM 2542 at the outset, but seemed to suffer growth inhibition which affected the rate of both glucose and xylose metabolism, and this coincided with the browning of the media.

### 3.3. Growth and sugar consumption of DSM 2542 *ptsH1* in the bioreactor

**Figure 2:**
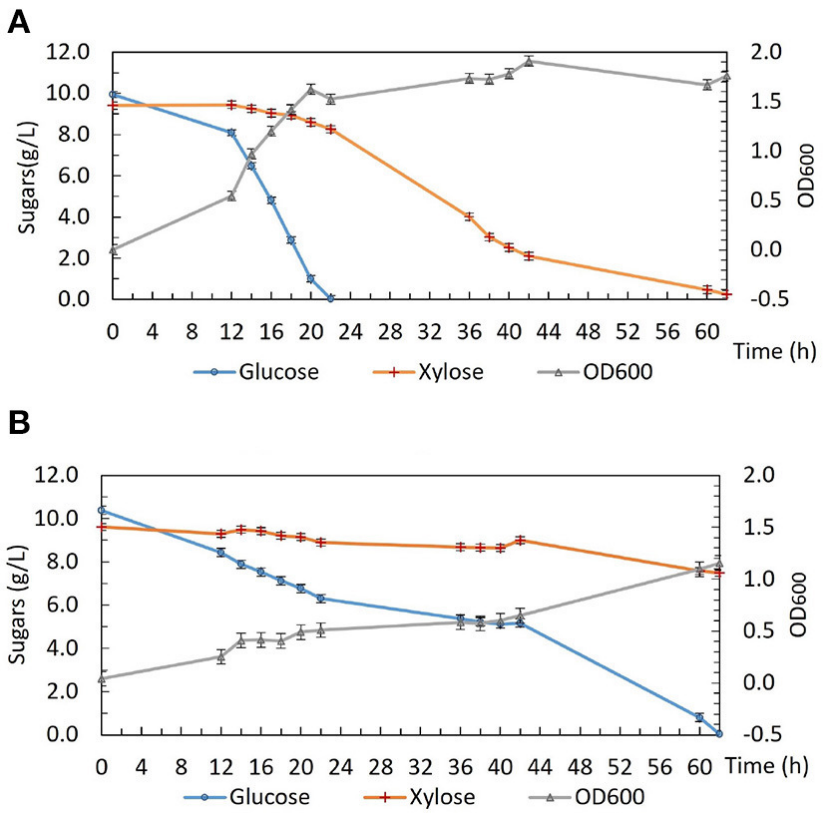
Growth and sugar consumption profile of **(A)** *P. thermoglucosidasius* DSM 2542 and **(B)** *P. thermoglucosidasius* DSM 2542 *pstH1* in ASM medium supplemented with 1% (w/v) glucose and 1% (w/v) xylose under fermentative conditions in a bioreactor. Error bars are ± standard deviation of three replicates taken at the same time point.

To investigate the phenotype of DSM 2542 *ptsH1* in more detail, growth studies were carried out in a bioreactor containing a glucose and xylose mixture under fermentative conditions. As shown in Figure 2, DSM 2542 *ptsH1* grew poorly compared with the wild type, consistent with the much slower sugar utilization observed in the tube fermentations. Indeed, unlike the tube cultures, which start with a period of aerobic growth, it is clear that the rate of glucose metabolism was restricted from an early stage in the culture. Nevertheless, DSM 2542 *ptsH1* still showed clear evidence of CCR as xylose metabolism did not increase to compensate. Within 22 h, the wild type reached an OD_600_ of about 1.5 with complete consumption of glucose. After that, it started to consume xylose and was completed in about 60 h. The total biomass did not increase much when it was growing on xylose, consistent with its low net ATP yield under anaerobic conditions. In contrast, DSM 2542 *ptsH1* hardly managed to metabolize the glucose within 60 h, and there was still a lot of xylose remaining after 86 h incubation. As seen during tube fermentation, a dark-brown color developed in the DSM 2542 *ptsH1* culture media within 12 h. Given the unexpected phenotype, the DSM 2542 *ptsH1* variant was sent for whole genome sequencing (MicrobesNG, Birmingham, UK), but this was confirmed that there were no mutations other than that made in *ptsH*, compared to the parental strain. Therefore, the slow growth and slow sugar utilization of DSM 2542 *ptsH1* under fermentative conditions must be the direct result of this mutation.

### 3.4. Growth and sugar consumption of DSM 2542 *ptsH1* under aerobic conditions

**Figure 3:**
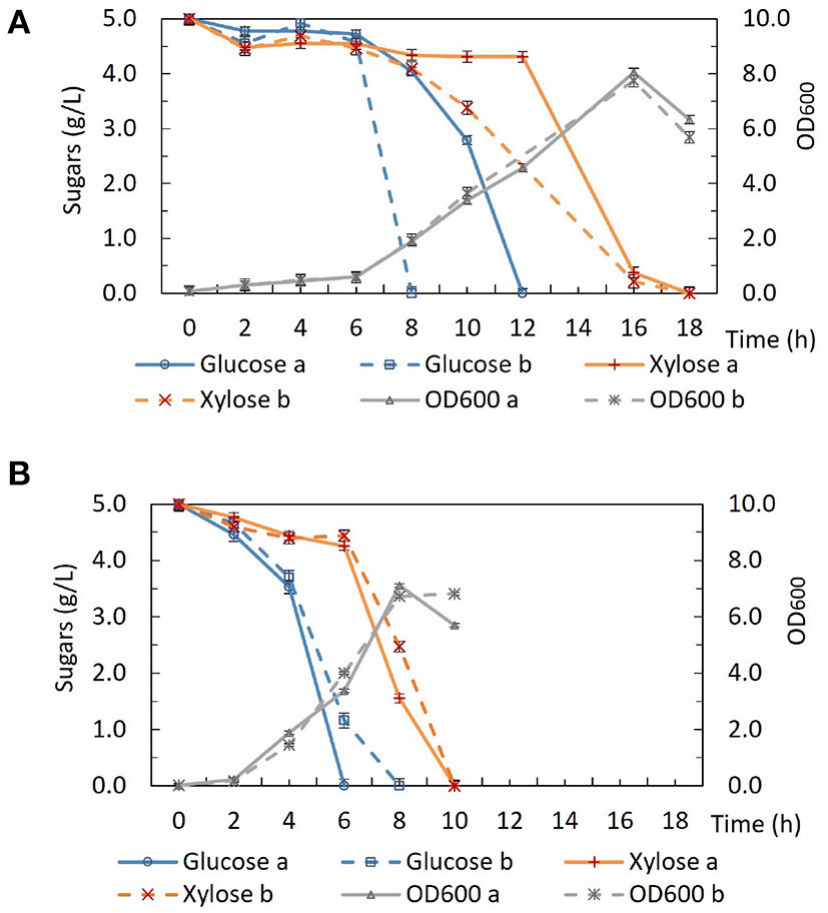
Growth and sugar consumption profile of **(A)** wild type *P. thermoglucosidasius* DSM 2542 and **(B)** *P. thermoglucosidasius* DSM 2542 *pstH1* in ASM medium supplemented with 0.5% (w/v) glucose and 0.5% (w/v) xylose under aerobic conditions. Two biological repeats are displayed in each graph using solid and dotted lines, respectively. Error bars are ± standard deviation of three technical replicates taken at the same time point.

Interestingly, unlike the situation under fermentative conditions, the growth of *DSM 2542 ptsH1* under aerobic conditions was similar to that of the wild type (Figure 3). Aerobic growth was performed in 50 mL ASM medium in 250 mL baffled Erlenmeyer flasks sealed with silicone sponges. The cultures were incubated in a shaking incubator (60°C, 250 rpm) and samples were taken out regularly for biomass and HPLC analysis. Under aerobic conditions, both DSM 2542 *ptsH1* and the wild type grew rapidly and reached a similar final OD_600_. Intriguingly, DSM 2542 *ptsH1* appeared to have a shorter lag phase and more rapid growth compared with the wild type. However, glucose was consumed prior to xylose in both strains, indicating that the *ptsH1* mutation did not relieve the cells from CCR under aerobic conditions either. Although the repeat of wild-type DSM 2542 both showed rapid growth on glucose there was a difference in the length of the lag phase, leading to a significant difference in glucose utilization after 6–12 h. Variability in the length of the lag phase is consistently observed with *P. thermoglucosidasius* DSM 2542 and NCIMB 11955, probably caused by a slight variation in the state of the inoculum. Notably, the dark-brown-color culture medium observed during fermentation was not observed under aerobic conditions. This experiment was repeated to confirm the results.

### 3.5. Identification of methylgloxal in *P. thermoglucosidasius* DSM 2542 and DSM2542 *ptsH1* cultures

**Figure 4:**
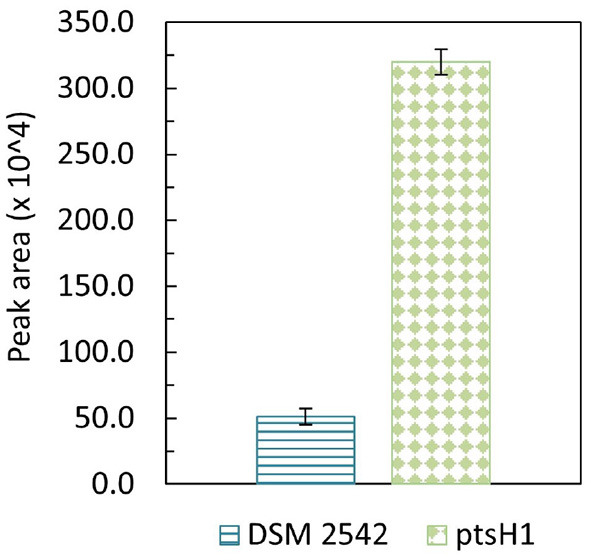
GC-MS peak area (m/z = 144) of methylglyoxal derivatised with 1,2-diaminobenzene from *P. thermoglucosidasius* DSM 2542 and DSM2542 *ptsH1* cultures after 48-h tube fermentation in ASM medium containing 1% (w/v) glucose and 1% (w/v) xylose. Error bars are standard deviation of three biological replicates. *T*-test confirmed that the peak areas were statistically different (*p* < 0.05).

Formation of a brown pigment resulting from the reaction of reducing sugars and amines (the Maillard reaction) is well-understood in microbiology and food science. This is known to be accelerated at high temperatures which is why it is advisable not to autoclave sugars with rich media containing amino acids. However, it is clear that the color formation was only happening with DSM 2542 *ptsH1*, suggesting metabolic production of a reactive aldehyde/ketone, which is more reactive than reducing sugars. The most likely candidate is methylglyoxal, which can be produced from the glycolytic intermediate dihydroxyacetone phosphate (DHAP) as a glycolytic bypass and is known to be a reactive compound which can contribute to similar browning reactions (Gandhi et al., 2019). To confirm the identity of this product, it was reacted with 1,2-diaminobenzene and the product analyzed by GC-MS. DSM 2542 and DSM2542 *ptsH1* were grown for 48 h in ASM medium (1% (w/v) glucose and 1% (w/v) xylose) under tube fermentation. As control, 10 μL methylglyoxal was added to 10 mL of the same ASM medium, followed by the derivatization and extraction. Both control and experimental groups showed a unique TIC peak at 10.68 min with MS principal ions at m/z = 117 and 144. Quantification was based on the intensity of principal ion m/z = 144 and the peak area of DSM2542 *ptsH1* was about 6 times higher than DSM 2542, indicating an elevated level of methylglyoxal excreted by DSM2542 *ptsH1* during fermentation (*p* < 0.05) (Figure 4).

The reaction between methylglyoxal and a 1,2-diamine in cell culture medium is a competitive equilibrium between the diamine and amino groups in the medium, so accurate quantification is difficult. Therefore, to get an approximate measure of concentration, known concentrations of methylglyoxal were added to DSM 2542 cultures grown to OD_600_ about 1.0 in ASM medium supplemented with 1% (w/v) glucose and 1% (w/v) xylose under fermentative conditions. After 3 h of incubation at 60°C, different shades of light-yellow color started to develop in all methylglyoxal-treated samples. After overnight incubation, the brown coloration observed in DSM 2542 *ptsH1* cultures was evident in the higher concentration groups (Supplementary Figure 3A), and after 36 h of incubation, all treated-groups turned into a gradient of dark-brown color (Supplementary Figure 3B), while the untreated group remained the normal beige color commonly seen in DSM 2542 fermentation using ASM medium (Supplementary Figure 3C). The browning effects caused by 0.1–0.2% (v/v) methylglyoxal were comparable to the color observed during the fermentation of DSM 2542 *ptsH1* after 36 h (Supplementary Figure 3D). Although ASM is not supplemented with amino acids the methylglyoxal could be reacting with secreted or lysis products.

### 3.6. Effects of 2-DG on *P. thermoglucosidasius* DSM 2542 growing on xylose

**Figure 5:**
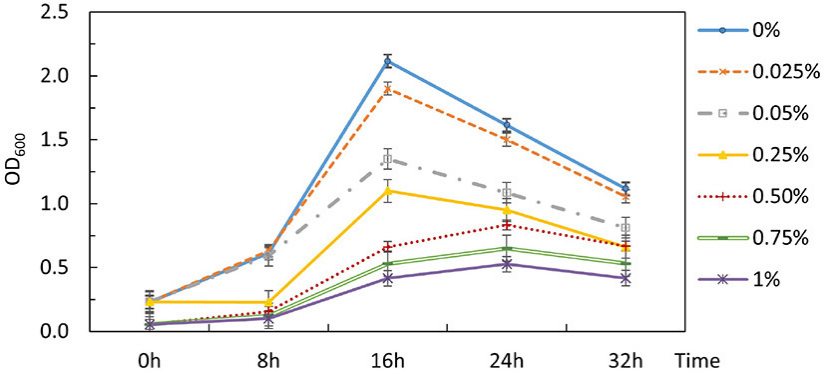
Effects of 2-DG during growth of *P. thermoglucosidasius* DSM 2542 on xylose in 50 mL conical centrifuge tubes. *P. thermoglucosidasius* DSM 2542 was grown in ASM medium supplemented with 1% (w/v) xylose and different concentrations of 2-DG (% w/v shown on the right). Error bars are ± standard deviation of three technical repeats.

The inability to alleviate CCR by removing the signaling components from Hpr or Hpr and Crh together necessitated an alternative strategy. The potential for 2-DG to act as a classical non-metabolizable glucose analog capable of exerting catabolite repression in *P. thermoglucosidasius* DSM 2542 was examined by including different concentrations of 2-DG (0-1% w/v) in an ASM medium supplemented with 1% (w/v) xylose. The overall growth rate decreased as 2-DG concentration increased (Figure 5). Extensive studies with *P. thermoglucosidasius* NCIMB 11955, which is virtually identical to DSM 2542, have shown that it typically does not enter an extended stationary phase, but starts to lyse after nutrient starvation. This pattern was also evident with DSM 2542 growing in tubes on xylose, where the OD_600_ (in the absence of 2-DG) peaked after 16 h. In the presence of 2-DG, the peak OD_600_ was lower and appeared later as 2-DG concentration increased. When supplemented with 0.5%(w/v) 2-DG, the peak OD_600_ was reduced by about a third and the growth rate was reduced by nearly 50%. This concentration was, therefore, used for adaptive evolution experiments.

### 3.7. Isolation and analysis of catabolite de-repressed mutant strains

**Figure 6:**
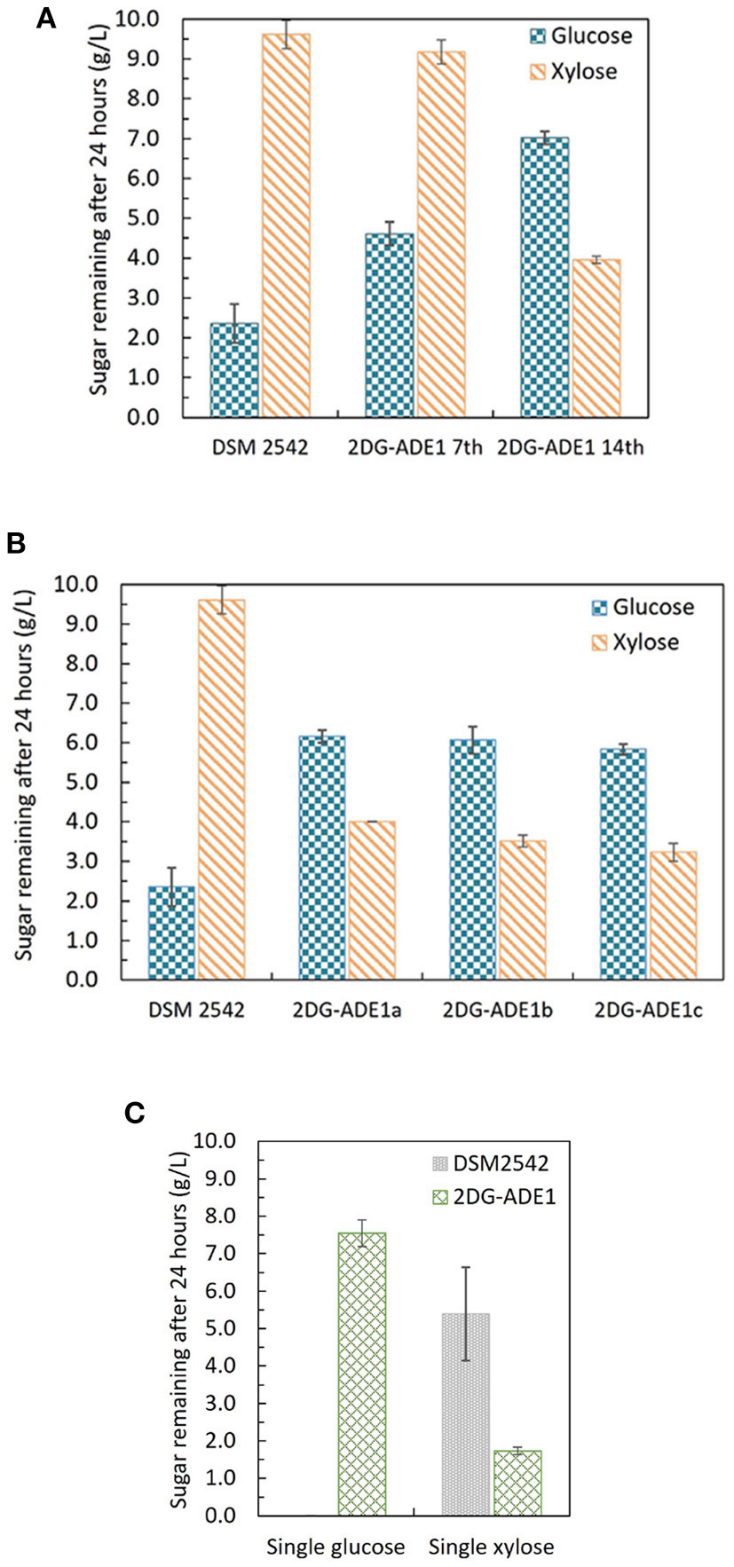
Sugar consumption profiles of *P. thermoglucosidasius* DSM 2542, 2-DG resistant cultures after 7 and after 14 passages in 0.5% 2-DG and 1% xylose (2DG-ADE1 7th and 2DG-ADE1 14th), and 2-DG resistant isolates 2DG-ADE1a, 2DG-ADE1b and 2DG-ADE1c during tube fermentation in 15 mL sterile centrifuge tubes. Cells were cultured in ASM medium supplemented with 1% (w/v) glucose and 1% (w/v) xylose as in **(A,B)**, or **(C)** 1% (w/v) glucose or 1% (w/v) xylose alone. To stabilize the pH during the fermentation, 0.2 g MgCO_3_ was added to the tubes prior to inoculation. Error bars in **(A,B)** and DSM 2542 from **(C)** are ± standard deviation of three replicates taken at the same time point. Error bars in **(C)** 2DG-ADE1 are ± standard deviation of 2DG-ADE1a, 2DG-ADE1b and 2DG-ADE1c samples taken at the same time point. A *t*-test performed on data in **(A,B)** confirmed the remaining glucose and xylose in cultures of DSM 2542 and the 2DG resistant strains were statistically different (*p* < 0.05).

*P. thermoglucosidasius* DSM 2542 grown in ASM medium supplemented with 1%(w/v) xylose and 0.5%(w/v) 2-DG was sub-cultured every 24 h. After 7 sub-cultures, the cell population was tested and found to still exhibit catabolite repression (Figure 6A). After 14 subcultures, the cell population was tested again and found to be catabolite de-repressed (Figure 6A). After growing on 2TY plates, three colonies (2DG-ADE1a,b, and c) were randomly picked from a total of about 100 for further characterization using tube fermentation in ASM medium supplemented with 1% (w/v) glucose and 1% (w/v) xylose (Figure 6B).

In the culture medium of the parent strain DSM 2542, about 27% of the glucose remained after 24 h, while 95% of xylose was unused due to CCR. In contrast, in the culture medium of 2DG-ADE1a, 2DG-ADE1b, and 2DG-ADE1c, about 57–60% of the glucose and 30–40% of the xylose remained after 24 h. Thus, while it appeared that glucose and xylose were being co-utilized by 2DG-ADE1a, 2DG-ADE1b and 2DG-ADE1c, indicative of catabolite de-repression, this seemed to be as a result of compromising glucose utilization. This was confirmed by growing each of the strains in tube fermentation in ASM medium supplemented with 1% (w/v) glucose or 1% (w/v) xylose alone (Figure 6C). When grown on glucose as sole carbon source, DSM 2542 used all the glucose within 24 h, while an average of 55% of glucose remained in the culture medium of 2DG-ADE1a, 2DG-ADE1b, and 2DG-ADE1c. When cultured on xylose alone, 75 and 18% of the xylose was left in the culture medium of DSM 2542 and these evolved strains, respectively, after 24 h, indicating that not only had glucose utilization been inhibited but xylose utilization had been enhanced.

To understand the genetic changes responsible for this phenotype, the genome sequences of five evolved strains isolated from the same culture (2DG-ADE1a, b, c, d and e) were compared with that of their parental strain. In total five mutations were identified by single nucleotide polymorphism (SNP) analysis, four of which were found in all five strains. Two of these were located in non-coding regions, and two were identified within coding regions (Supplementary Table 1): an alanine to glutamate substitution at position 19 (A19E) on phosphoenolpyruvate-protein phosphotransferase PtsI (also called enzyme I or EI) encoded by *ptsI*, and a phenylalanine to valine substitution at position 74 (F74V) on adenine phosphoribosyl transferase (APRT) encoded by *apt*. These potential influence of these two mutations on sugar utilization will be discussed later.

### 3.8. Isolation and analysis of 2-DG resistant strains which retain rapid growth on glucose

**Figure 7:**
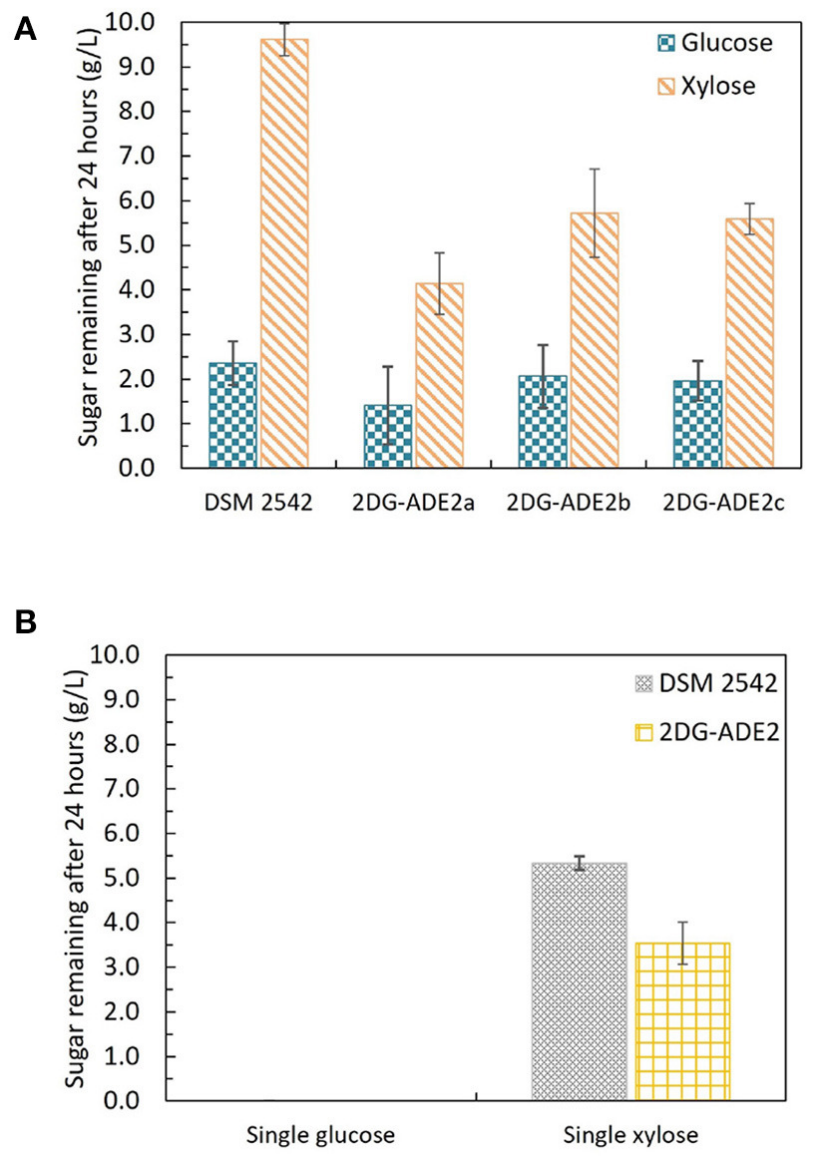
Sugar consumption profiles of *P. thermoglucosidasius* DSM 2542 and 2-DG resistant strains 2DG-ADE2a, 2DG-ADE2b and 2DG-ADE2c during tube fermentation in 15 mL sterile centrifuge tubes. Cells were cultured in ASM medium supplemented with **(A)** 1% (w/v) glucose and 1% (w/v) xylose or **(B)** 1% (w/v) glucose or 1% (w/v) xylose. To stabilize the pH during the fermentation, 0.2 g MgCO_3_ was added to the tubes prior to inoculation. Error bars in **(A,B)** DSM 2542 are ± standard deviation of three replicates taken at the same time point. Error bars in **(B)** 2DG-ADE2 are ± standard deviation of 2DG-ADE2a, 2DG-ADE2b, and 2DG-ADE2c samples (three replicates) taken at the same time point. A *t*-test performed on data in **(A)** confirmed the remaining glucose and xylose in each strain were statistically different (*p* < 0.05).

The first adaptive evolution experiment clearly generated CCR-negative strains but at the expense of rapid glucose metabolism, which is not useful for industrial fermentation. An improved experimental design was, therefore, required to prevent impaired glucose utilization becoming a selected phenotype during the evolutionary process. To achieve this an intermediate selection step for good growth on glucose was required. Similar to the first evolution experiment, DSM 2542 was initially grown in 1% (w/v) xylose and 0.5% (w/v) 2-DG and sub-cultured every 24 h. Then, after 7 subcultures, the cell population was enriched for strains with rapid growth on glucose, followed by a further 7 subcultures in the repressive medium containing 2-DG and xylose. Finally, the cell population was tested for evidence of catabolite de-repression during tube fermentation on a mixture of glucose and xylose before plating out on 2TY plates. Three colonies (2DG-ADE2a, b, and c) were randomly selected from about 100 colonies on the 2TY plates for further characterization. During tube fermentation in ASM medium supplemented with 1% (w/v) glucose and 1% (w/v) xylose, compared with DSM 2542, xylose was more extensively metabolized by 2DG-ADE2a, 2DG-ADE2b and 2DG-ADE2c than by DSM 2542 after 24 h, suggesting xylose consumption in these strains was de-repressed (Figure 7A). However, unlike the strains from the first evolution experiment, 2DG-ADE2a, 2DG-ADE2b and 2DG-ADE2c utilized similar amounts of glucose to the wild type DSM 2542 in the first 24 h. When grown in ASM medium supplemented with 1% (w/v) glucose alone, they were able to metabolize all the glucose within 24 h like DSM 2542, so their rate of glucose utilization did not seem to be compromised (Figure 7B). Similar to the strains from the first evolution, during growth in ASM medium supplemented with 1% (w/v) xylose alone, they consumed more xylose than DSM 2542 in the first 24 h, suggesting their rate of xylose utilization had increased (Figure 7B). However, this enhancement was not as great as that in the strains from the first evolution experiment.

To understand the genetic changes responsible for such phenotypes, the genome sequences of five evolved strains from the same culture (2DG-ADE2a, b, c, d, and e) were compared with that of their parental strain DSM 2542. In total 43 SNPs were identified, many within non-coding regions, although 8 of those SNPs were found in coding regions of all five strains (Supplementary Table 2). Notably, in all of them, a stop codon was introduced at Q10 in the *mutS* gene. The *mutS* gene encodes a DNA mismatch repair protein which is involved in the repair of errors in DNA replication which causes mismatches (Wang et al., 2003). Termination of *mutS* translation would significantly increase the mutation rate, so if this had been an early evolutionary event this could explain the high frequency of SNPs in these evolved strains. Among the common mutations within coding regions, three were directly linked to sugar metabolism: one was an arginine to cysteine substitution at position 452 (R452C) on PTS glucose transporter subunit IICBA (PtsG or EIICBA^*Glu*^) encoded by *ptsG*, one was a frame-shift after the threonine at position 58 on the ribose operon repressor (RbsR) encoded by *rbsR* gene, and the other one, interestingly, was the same APRT-F74V mutation which appeared in the first evolution experiment. The SNPs identified in these genes were confirmed by Sanger sequencing of PCR products.

**Figure 8:**
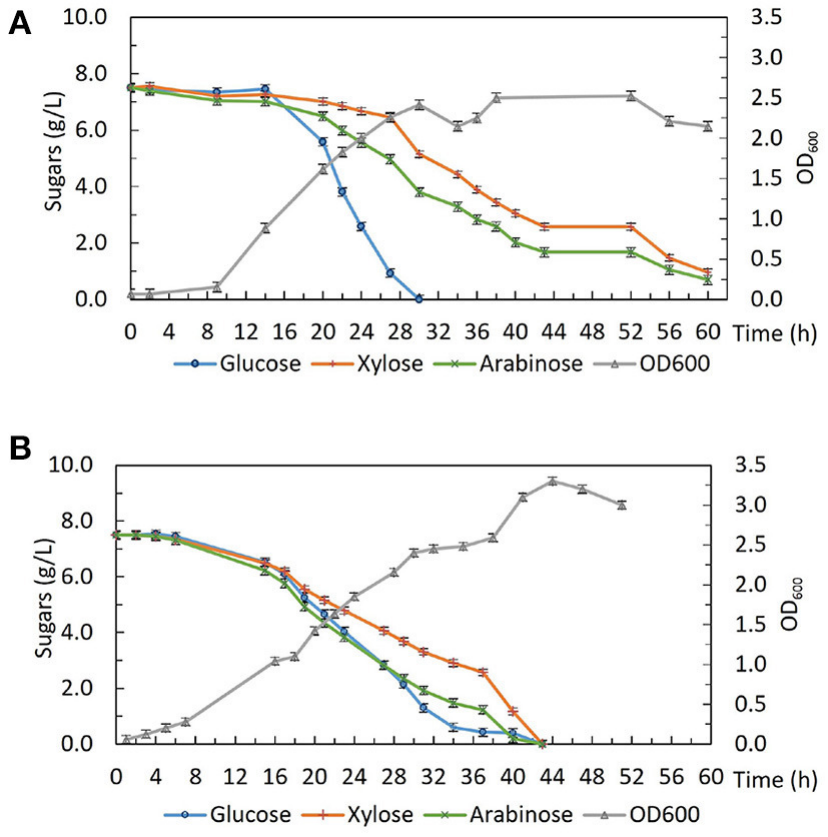
Fermentative growth and sugar consumption profile of **(A)** *P. thermoglucosidasius* DSM 2542 and **(B)** 2-DG resistant strain 2DG-ADE2b in ASM medium supplemented with 0.75% (w/v) glucose, 0.75% (w/v) xylose and 0.75% (w/v) arabinose in bioreactors. Error bars are ± standard deviation of three replicates taken at the same time point.

Strains from this evolution experiment showed signs of catabolite de-repression as well as enhanced xylose utilization without compromising glucose consumption, so in order to characterize their growth and sugar consumption profiles under more controlled conditions. 2DG-ADE2b, which contained the fewest SNPs among the five sequenced strains, was compared with the wild type DSM 2542 in bioreactors under fermentative conditions. Bench-top bioreactor operation, as well as HPLC analysis of samples taken at regular intervals, were as described previously (Liang et al., 2021). The growth medium contained glucose, xylose and arabinose which are the main sugars in lignocellulosic hydrolysates. Figure 8A shows that, due to CCR, DSM 2542 had a clear preference for glucose, metabolizing it in 30 h and, reaching a peak OD_600_ of about 2.5. During this period, a small amount of xylose and arabinose were consumed because the CCR in *P. thermoglucosidasius* is not as tightly regulated as in some other organisms (Liang et al., 2021). After glucose had been exhausted there was a transient drop in OD_600_ associated with the switch in carbon sources but the OD_600_ effectively plateaued during the subsequent period of metabolism of xylose and arabinose. In contrast, 2DG-ADE2b utilized glucose, xylose and arabinose simultaneously, and reached a higher peak OD_600_ of about 3.4 (Figure 8B). DG-ADE2b metabolized all the sugars within 42 h, so the total fermentation time was shorter, compared to DSM 2542. This experiment was repeated and the same trend was observed. These results collectively indicate that removal of CCR has a positive impact on fermentation providing a more controlled metabolic environment. The rate of total sugar metabolism (qs) was 0.003 mol/gDW/h in 2DG-ADE2b and 0.002 mol/gDW/h in DSM 2542, which were similar, indicating there may be a maximum metabolic flux from carbohydrates into central metabolism.

### 3.9. Effect of complementation with *ptsG, rbsR*, and *apt* on CCR in the evolved strain

To understand which mutation(s) contributed to the removal of CCR from *P. thermoglucosidasius* DSM 2542, genetic complementation was used to investigate whether the wild type *ptsG, rbsR* and *apt* genes would restore CCR in 2DG-ADE2b. 2DG-ADE2b was transformed separately with plasmid pJL-*ptsG*^+^, pJL-*apt*^+^ and pJL-*rbsR*^+^, resulting in 2DG-ADE2b variants capable of expressing either *ptsG* or *rbsR* or *apt* from their own promoters (Supplementary Figure 4).

**Figure 9:**
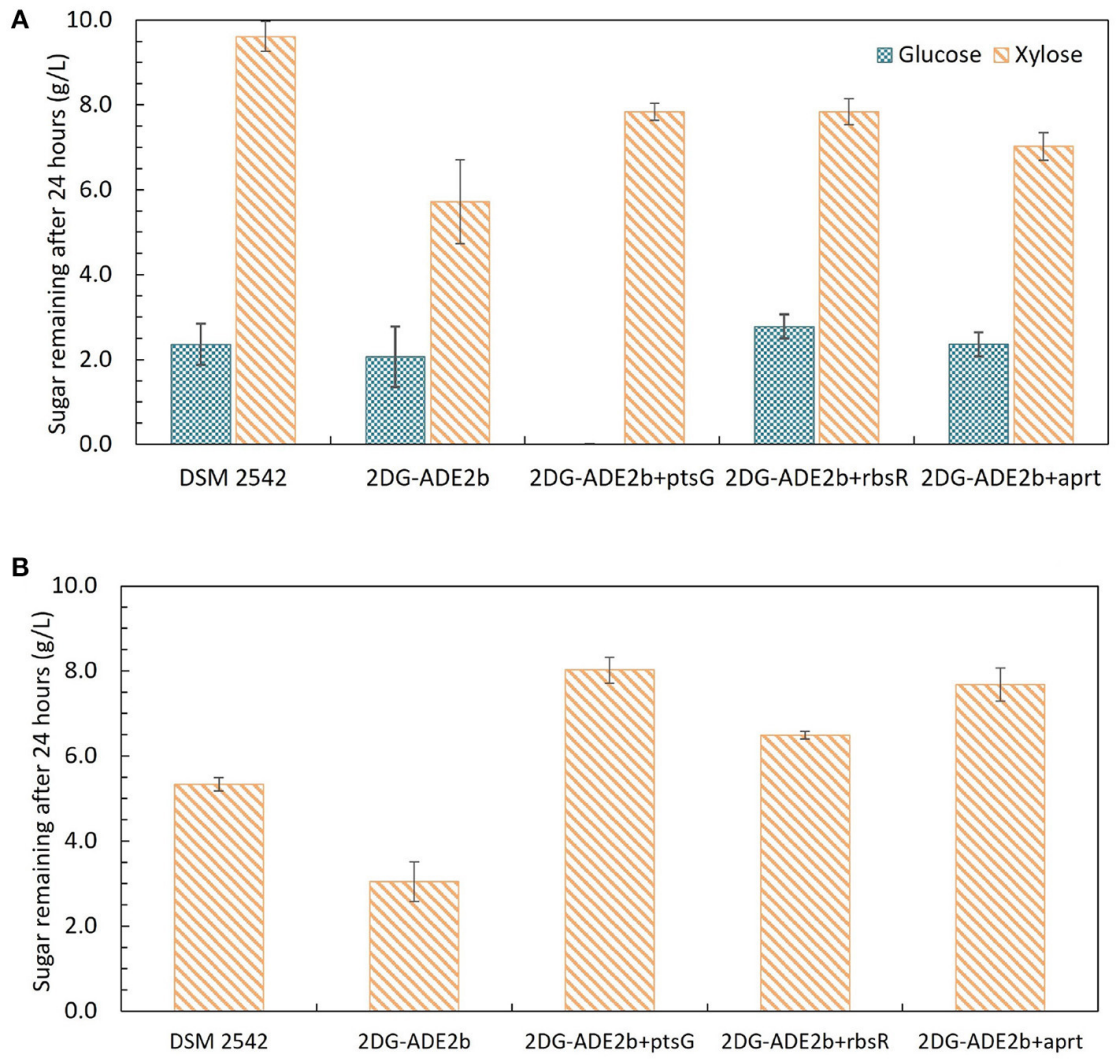
Sugar consumption profiles of *P. thermoglucosidasius* DSM 2542, 2-DG evolved strains 2DG-ADE2b, and 2DG-ADE2b variants with plasmid pJL-*ptsG*^+^(2DG-ADE2b*ptsG*^+^), pJL-*apt*^+^ (2DG-ADE2b*apt*^+^) and pJL-*rbsR*^+^ (2DG-ADE2b*rbsR*^+^), respectively. Cells were grown in 15 mL sterile centrifuge tubes in ASM medium supplemented with **(A)** 1% (w/v) glucose and 1% (w/v) xylose, or **(B)** 1% (w/v)xylose. To stabilize the pH during the fermentation, 0.2 g MgCO_3_ was added to the tubes prior to inoculation. Error bars are ± standard deviation of three replicates taken at the same time point. *T*-test confirmed the remaining glucose and xylose in each culture in **(A)** were statistically different (*p* < 0.05), and the remaining xylose in **(B)** was statistically different from each other (*p* < 0.05).

Growth of these variants under different sugar conditions was compared using tube fermentation (Figure 9). When they were grown in ASM medium supplemented with 1% (w/v) glucose as the sole carbon source, similar to the wild type DSM 2542 and 2DG-ADE2b, they finished all glucose within 24 h (data not shown). However, when they were grown in ASM medium supplemented with a mixture of 1% (w/v) glucose and 1% (w/v) xylose, or 1% (w/v) xylose as the sole carbon source, they all consumed xylose more slowly than 2DG-ADE2b. When the *ptsG* complemented variant, 2DG-ADE2b*ptsG*^+^, was grown in ASM medium supplemented with 1% (w/v) glucose and 1% (w/v) xylose, it consumed all of the glucose in the first 24 h, while 80% of the xylose remained, typical of a cell exhibiting catabolite repression. However, when it was grown in 1% (w/v) xylose as the sole carbon source, there was still 80% of unconsumed xylose after 24 h as in the mixed-sugar substrate, suggesting that complementation with *ptsG* on a plasmid was inhibiting xylose utilization. In contrast, the wild type DSM 2542 consumed both less xylose and less glucose when the two substrates were combined due to CCR, but it consumed xylose on its own more rapidly than the complemented strain. Clearly, the effect of *ptsG* complementation is not straightforward, but it should be noted that the use of a plasmid-borne gene would have increased the gene dosage and hence, the expression level of PtsG.

When 2DG-ADE2b*apt*^+^ and 2DG-ADE2b*rbsR*^+^ were grown in 1% (w/v) glucose and 1% (w/v) xylose, the concentration of glucose remaining after 24h was similar to cultures of DSM2542 or 2DG-ADE2b, however the concentration of xylose remaining unconsumed was less than observed with DSM2542, but more than 2DG-ADE2b indicating a partially repressed phenotype. However, as with the *ptsG* complemented strain, when they were grown in 1% (w/v) xylose alone, more xylose remained in the culture medium after 24 h compared with DSM 2542 or 2DG-ADE2b. These results suggested that *apt*^+^ and *rbsR*^+^ expressed on a plasmid might restrict xylose uptake or utilization, but they might not directly cause CCR in DSM 2542.

## 4. Discussion

In *B. subtilis*, CCR of several genes can be relieved by replacing the Ser46 residue of both HPr and Crh with an Ala, while a single S46A mutation on HPr or Crh was not sufficient to remove CCR (Galinier et al., 1999; Puri-Taneja et al., 2006). Similarly, neither the *ptsH1* nor the *crh1* mutation alone eliminated CCR in *P. thermoglucosidasius* DSM 2542, but multiple attempts to isolate a *ptsH1 crh1* double mutant were not successful, using a method which consistently yields mutants in non-essential genes. This suggests that the double mutation is highly deleterious or lethal in *P. thermoglucosidasius* DSM 2542 promoting a search for an alternative strategy. In principle, this could have involved modification of downstream components of the CCR signaling pathway, such as deletion/mutation of *ccpA* or modification of *cre* sites (Kraus et al., 1994; Miwa et al., 1997; Nathan and Nair, 2013). However, previous studies suggested that deletion of *ccpA* was also deleterious/lethal (Holland, 2017), and modification of *cre* sites would have to be done for each repressible substrate. Hence, we reverted to a traditional substrate analog approach, which proved to be simple and remarkably effective.

### 4.1. Formation of HPr (Ser-P) is necessary to control MgsA activity under fermentative conditions

**Figure 10:**
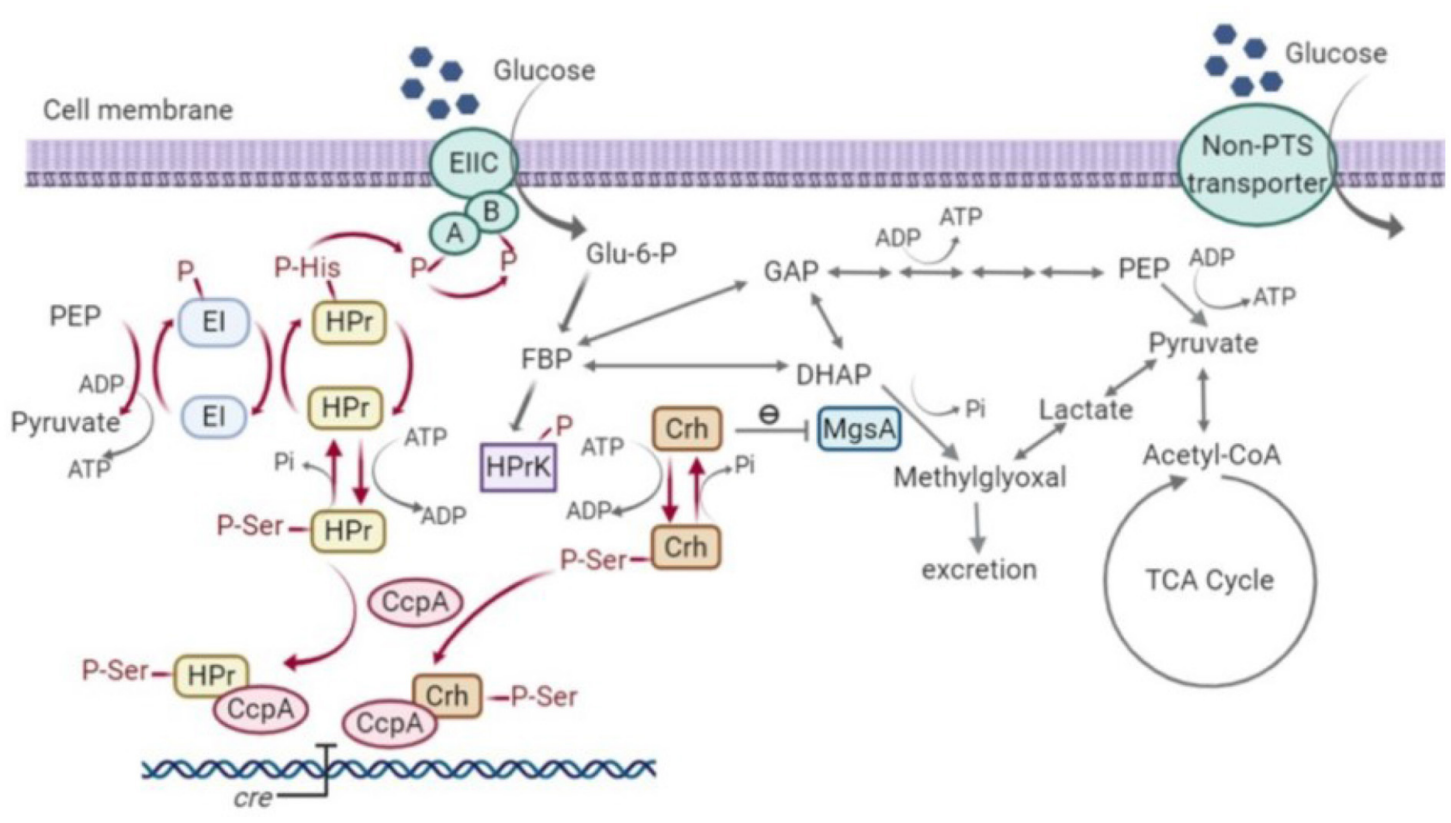
Signal transduction pathway of carbon catabolite repression (CCR) and methylglyoxal pathway in *Bacillus subtilis* (Görke and Stülke, 2008; Fujita, 2009; Landmann et al., 2011). HPr, phosphocarrier protein; Crh, catabolite repression HPr; CcpA, carbon catabolite protein A; HPrK, HPr kinase; MgsA, methylglyoxal synthase; GAP, glyceraldehyde-3-phosphate; DHAP, dihydroxyacetone phosphate; EI, Enzyme I; EII, Enzyme II; PEP, phosphoenolpyruvate; Glu-6-P, glucose-6-phosphate; FBP, fructose-1,6-bisphosphate; Acetyl-CoA, acetyl-coenzyme A; TCA, tricarboxylic acid cycle; *cre*, catabolite-response element.

As well as the inability to create a *ptsH1 crh1* double mutant, the single *ptsH1* mutant was shown to have a novel phenotype under fermentative, but not aerobic conditions, namely the production of methylglyoxal, presumably resulting from the over-activation/upregulation of methylgloxal synthase (MgsA). The fact that this was observed under fermentative conditions alone may explain why this has not been observed with *B. subtilis*, where most studies have focussed on aerobic growth, as the ability of the phosphorylation state of Crh to control the activity of MgsA has been established in that host (Landmann et al., 2011). Crh participates in glycolytic flux regulation by interacting with MgsA. MgsA produces methylglyoxal from dihydroxyacetone phosphate (DHAP) and is either excreted and/or converted to lactate and pyruvate *via* various pathways (Landmann et al., 2011). The methylglyoxal pathway acts as a glycolytic bypass under carbon overflow conditions, in order to prevent the accumulation of phosphorylated glycolytic intermediates which are harmful to cells (Eisermann et al., 1988). However, methylglyoxal is toxic and therefore its synthesis is under tight regulation. Under nutritional famine conditions, Crh is typically in its non-phosphorylated state due to the low fructose-1,6-bisphosphate (FBP) concentration. The non-phosphorylated form of Crh inhibits MgsA activity and prevents the formation of methylglyoxal. However, under feast conditions, high FBP concentrations result in the phosphorylation of Crh by HPr kinase. Crh (Ser-P) cannot interact with MgsA and consequently, carbon flux is allowed to flow through the methylglyoxal pathway to relieve phospho-sugar stress (Figure 10) (Landmann et al., 2011).

The over-stimulation of MgsA resulting in methylglyoxal accumulation and toxicity seen in the current study indicates that formation of HPr (Ser-P) is also necessary to control MgsA activity. While it could reflect an over-production of Crh (Ser-P) due to lack of competition between Hpr and Crh for Hpr kinase, this phenotype was not seen in the Hpr deletion strain TM444 △*ptsH*, so it is specific to the phosphorylation state of Hpr. An alternative explanation is that HPr (Ser-P) competes with Crh (Ser-P) for its binding site on MgsA, thus modulating the level of stimulation. One caveat to that argument is that deletion of *ptsH* eliminates glucose transport through the PTS system, resulting in slower growth (surprisingly, this eliminates growth of *B. subtilis* on glucose, despite the ability to produce an alternative MFS glucose transporter) and, hence, less accumulation of phospho-sugar intermediates.

### 4.2. Analysis of key mutations in both adaptive evolution experiments

As an alternative strategy to isolate CCR resistant mutants, 2-DG was used as a non-metabolizable substrate analog to induce CCR, providing a platform for selection of CCR-resistant mutants in *P. thermoglucosidasius* DSM 2542. Sequencing of isolates which were able to utilize PTS and non-PTS sugars simultaneously, revealed that none of the mutations were in the key regulatory proteins HPr, Crh and CcpA which are regarded as key players in the CCR of Gram-positive bacteria. However, in both evolution experiments, mutations were observed in PTS components, consistent with the observation that CCR typically involves sugars which are transported by, and signaling from the PTS system. The PTS is one of the major carbohydrate transport systems in bacteria (Deutscher et al., 2014): the glucose-PTS components consist of an EI, the HPr protein, encoded by *ptsI* and *ptsH*, respectively, and an inner membrane permease EII. EI and HPr are sugar non-specific, while EII is glucose-specific (also known as EII^*Glu*^) (Stülke and Hillen, 2000). The EII^*Glu*^ in *B. subtilis* and *P. thermoglucosidasius* is comprised of a single polypeptide consisting of three domains (EIICBA^*Glu*^) encoded by *ptsG*, and sugar transport is actually performed by the membrane-bound EIIC^*Glu*^ domain during which glucose is phosphorylated (Stülke and Hillen, 2000; Uniprot Consortium, 2019). The EIIA^*Glu*^ (which is a separate polypeptide in some organisms, such as *E. coli*) and EIIB^*Glu*^ components form a phosphate transfer network for glucose translocation, in which phosphoenolpyruvate (PEP) serves as a phosphoryl donor (Deutscher, 2008). Initially, a phosphoryl group from PEP is transferred to EI, which subsequently transfers it to the catalytic His-15 residue of HPr. HPr-His15-P then donates the phosphoryl group to EII^*Glu*^, initially to the EIIA^*Glu*^ domain (Postma et al., 1993; Lorca et al., 2005). Within EII^*Glu*^, the phosphoryl group is transferred from EIIA^*Glu*^ to EIIBC^*Glu*^, which mediates glucose translocation by converting extracellular glucose to intracellular glucose-6-phosphate (Teplyakov et al., 2006). In this process, glucose is transported by EIIC^*Glu*^ and phosphorylated by EIIB^*Glu*^.

#### 4.2.1. PtsI-A19E

**Figure 11:**
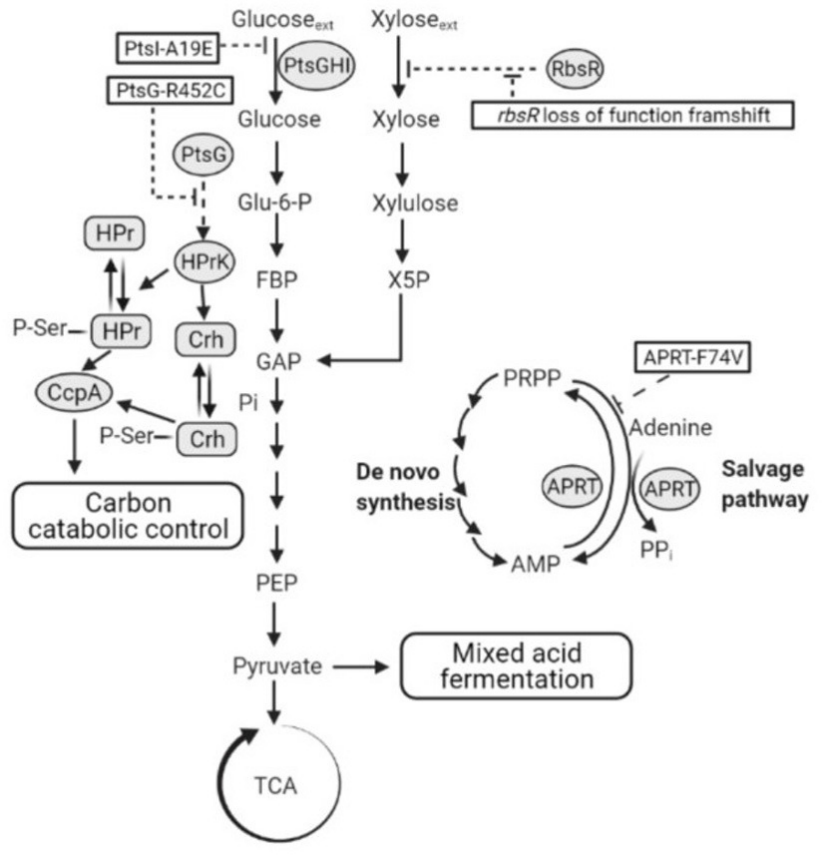
A schematic showing hypothetical impacts of PtsI-A19E, PtsG-R452C, and APRT-F74V mutations, and the *rbsR* loss of function frameshift mutation, respectively. Dotted lines are hypotheses proposed in this study. PtsGHI, glucose-specific phosphoenolpyruvate:sugar phosphotransferase system (PTS); RbsR, ribose operon repressor; TCA, tricarboxylic acid cycle; ACS, acetyl-CoA synthetase; APRT, adenine phosphoribosyl transferase; PRPP, adenine and phosphoribosyl pyrophosphate; AMP, adenosine monophosphate; CoA, coenzyme A; HPr, phosphocarrier protein HPr; Crh, HPr-like protein Crh; CcpA, catabolite control protein A; HPrK, HPr kinase.

In the first round of evolution in the presence of 2-DG, mutants were isolated in which the rate of glucose uptake under fermentative conditions was reduced and, in each isolate, sequenced the mutation PtsI-A19E (or EI-A19E) was present. EI is highly conserved among different bacteria, including that in *P. thermoglucosidasius* DSM2542, which has 78% sequence identity across its entire length to that from *B. subtilis* (NCBI Basic Local Alignment Search Tool). It consists of an N- and a C-terminal domain (EIN and EIC). The EIN has two subdomains connected by linkers; one is an EINα/β subdomain bearing the histidine active-site (His189 in *E. coli* and His189 in *B. subtilis*, His190 in *Staphylococcus carnosus* and probably His191 in *Staphylococcus aureus*), the other one is an EINα-helical subdomain which binds to HPr (Nosworthy et al., 1998; Márquez et al., 2006; Takayama et al., 2011). The EIC domain accepts a phosphoryl group from PEP and auto phosphorylates the histidine active site on the EINα/β subdomain. The phosphoryl group is then transferred to the HPr which binds to the EIN subdomain (Takayama et al., 2011). A previous study of *E. coli* EIN showed that the non-phosphorylated His189 active site is buried near the interface between the EINα/β and the EINα subdomains. Upon phosphorylation, EIN undergoes small conformational changes and the His189 rotates toward the surface, allowing for being approached by HPr-His-15 (Nosworthy et al., 1998). While not close to the active site, the A19E mutation is located within the EINα/β subdomain and substitution of a small non-polar alanine with a more bulky negatively charged glutamic acid residue could result is significant structural rearrangement, which clearly reduces or eliminates the activity of the EI protein (Figure 11). This loss of activity was also evident during growth on glucose alone and is consistent with a study in *Bacillus cereus*, which showed that deletion of the *ptsI* gene inhibited glucose uptake and utilization. The resulting phenotype is very similar to that of TM444 △*ptsH*, with slow glucose uptake failing to trigger CCR, so allowing activation of xylose uptake and metabolism. However, it is not a very useful phenotype for industrial exploitation as reduction in CCR results from impaired glucose uptake.

#### 4.2.2. PtsG-R452C

**Figure 12:**
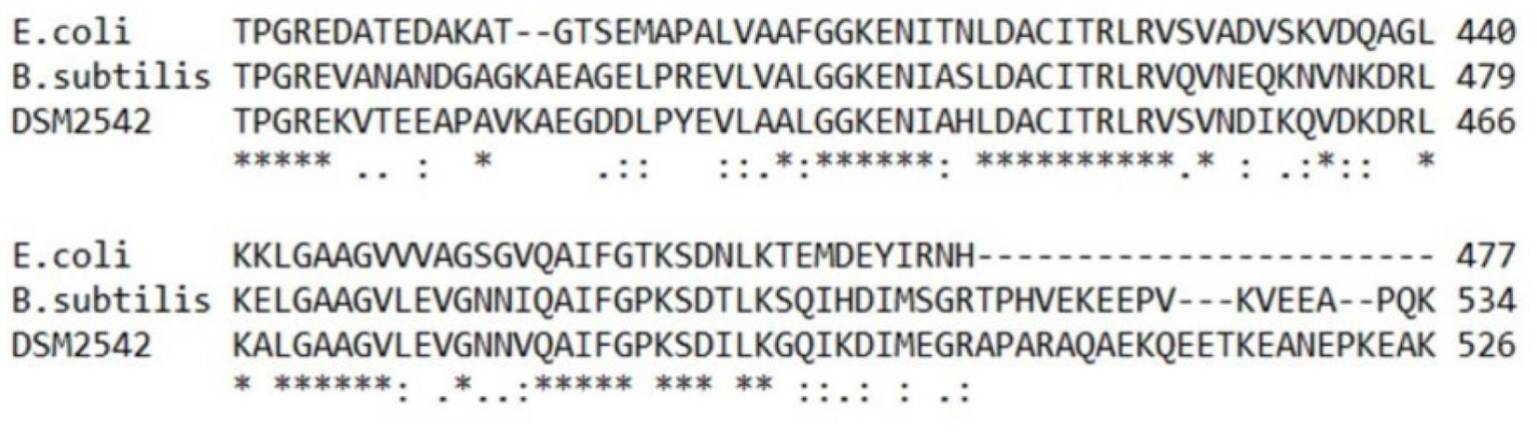
Amino acid sequence alignment of *E. coli* K-12, *B. subtilis* 168 and *P. thermoglucosidasius* DSM 2542 using UniProt Clustal Omega program. Regions around conserved sequence DACITRLR in PtsG are compared. * Indicates positions contain fully conserved residues,: indicates strongly similar properties, while. indicates weakly similar properties.

The PtsG-R452C (or EIIB^*Glu*^-R452C) mutation obtained in the second evolution is far more useful as this seemed to allow continued glucose uptake but reduced CCR. In *E. coli*, there are three essential motifs on EIIB^*Glu*^ within a strongly conserved sequence (DACITRLR) between amino acids Asp-419 and Arg-426, among which Cys-421 is the site of phosphorylation by EIIA^*Glu*^. Two invariant arginines (Arg-424 and Arg-426) stabilize the phosphate with their guanidino groups by forming hydrogen bonds and electrostatic interactions (Gutknecht et al., 1998; Seitz et al., 2003). Replacement of both Arg-424 and Arg-426 by lysines, produced EIIB^*Glu*^ mutants which were still able to accept the phosphoryl group from EIIA^*Glu*^, but could no longer transfer it to glucose, suggesting that these residues participated primarily in phospho-donor activity toward glucose but not in phospho-acceptor activity (Lanz and Erni, 1998). In *B. subtilis*, the conserved DACITRLR sequence was also found in EIIB^*Glu*^, with two arginines adjacent to the phosphorylated Cys-461 site (Bachem et al., 1997; Gutknecht et al., 1998). *P. thermoglucosidasius* DSM 2542 EIIB^*Glu*^ displays 73% amino acid sequence identity to the EIIB^*Glu*^ from *B. subtilis* (NCBI Basic Local Alignment Search Tool), has the conserved DACITRLR sequence located between Asp-445 and Arg-452 (Figure 12).

Surprisingly, the R452C mutation did not appear to impair glucose consumption in 2DG-ADE2b. A preliminary study showed that, when glucose was the sole carbon source during tube fermentation, 2DG-ADE2b consumed glucose at a similar rate to DSM 2542 (data not shown). However, a reduction in transfer rate from EIIB^*Glu*^ to glucose may not significantly affect growth if this step is not rate-limiting. Nevertheless, when multiple copies of the wild type *ptsG* were introduced to this strain the rate of glucose uptake and consumption increased, suggesting that the overall level of activity of EII was rate limiting, but this may reflect the transfer from EIIA^*Glu*^ to EIIB^*Glu*^, rather than EIIB^*Glu*^ to glucose. The fact that this mutation does not reduce the rate of glucose uptake implies that the phospho-sugar signals thought to give rise to CCR in the wild type should be present and that the R452C mutation somehow disrupts signal transduction more directly (Figure 11). Clearly, this mutation could affect the steady state degree of phosphorylation of EIIB^*Glu*^ by changing the relative rates of phosphate transfer between EIIA^*Glu*^ and glucose, so this could have revealed an important, previously unrecognized, CCR signal transduction mechanism.

Interestingly, the *ptsG* over-expression strain grew poorly on xylose as sole carbon source, which would be consistent with EII^*Glu*^ being involved in signal transduction. In the absence of glucose, EIIA^*Glu*^, EIIB^*Glu*^ at normal levels of expression would be expected to be phosphorylated. However, at high expression levels the rate of transfer of phosphate from HPr(H15)-P may not be sufficient to fully phosphorylate EII, resulting in a CCR “low phosphorylation state” signal being transmitted, even in the absence of glucose.

#### 4.2.3. Frame-shift in RbsR

As well as having global effects on expression, in Gram negative organisms, CCR also affects the transport of inducers of expression of repressible operons in a process of inducer exclusion. Analogous processes operate in Gram positive bacteria, e.g., in *B subtilis*, glucose has been shown to compete with xylose for binding to the XylR repressor, thus limiting expression of the *xylAB* operon, even in the presence of xylose. Genome analysis has revealed that *P. thermoglucosidasius* does not have genes encoding XylR or a typical xylose transporter, yet it is still able to grow with xylose as the sole carbon source. Therefore, xylose transport might be regulated and mediated by the activity of other pentose regulators and transporters, as has been described for *B. subtilis* (Krispin and Allmansberger, 1998).

In line with this, qRT-PCR results showed that the ribose transporter in DSM 2542 was up-regulated during growth on xylose, though not as much as during growth on ribose, suggesting that xylose is taken up by the ribose transporter (Liang, 2022). This suggestion is supported by the presence of the frame-shift mutation in *rbsR* in all of the CCR de-repressed 2DG-ADE mutants which, notably all metabolized xylose more rapidly than the wild-type when it was a sole carbon source (Figure 11). Loss of the RbsR would result in uncontrolled expression of the ribose transporter which might fortuitously transport xylose, but is not direct evidence that *P. thermoglucosidasius* DSM2542 has evolved to use it in the absence of a dedicated xylose transporter. However, the fact that the 2DG-ADE2b strain over-expressing a functional rbsR grew more slowly that both the wild-type DSM2542 on xylose alone, strongly suggests that the high levels of RbsR are out-competing the xylose. Together with the qRT-PCR evidence and the fact that this mutation did not appear in *araR*, it is reasonable to conclude that xylose can both induce and be transported by the ribose transporter in *P. thermoglucosidasius* DSM2542.

#### 4.2.4. Aprt-F74V

Finally, the Aprt-F74V mutation also seems to be important in evolved resistance to 2-DG and over-expression of a wild-type version of this gene showed the same effects as *ptsG* and *rbsR* in partially restoring CCR (although to a lesser extent) but also reducing the rate of xylose metabolism when xylose was the sole substrate. Mutations in Aprt, and in particular the Aprt-F74V mutation appeared in both evolution experiments and have also been reported in an earlier evolutionary study which was not focussing on CCR (Zhou et al., 2016). In the latter, 3 different mutations were found including a mid-gene frame-shift mutation; deletion of *aprt* was shown to have a similar phenotype of improved ethanol production from glucose but dependent on the addition of acetate. The association with CCR is unclear and arguments presented previously should not hold true here; based on the structure of the similar sized yeast enzyme, F74 is in a relatively conserved region at the homo-dimer interface and forms an inter-chain backbone (O-N) hydrogen bond. So a relatively conservative (hydrophobic-hydrophobic) mutation seems unlikely to disrupt dimer formation and is more likely to have a subtle effect on activity, possibly *via* the hinge region to the catalytic loop, which moves over the active site during catalysis. If the effect was to inactivate Aprt then it is surprising to find the same mutation appearing in 3 independent experiments (and exclusively in the two presented here). The characterized function of Aprt is to scavenge adenine from nucleotide degradation, although the reaction is reversible and recent studies with the human enzyme have shown that the conserved tyrosine in the catalytic loop is essential for driving the reaction in the forward (AMP synthesizing) direction (Huyet et al., 2018). As a more energy efficient pathway than *de-novo* synthesis, the purine salvage pathway is clearly important during growth, as shown by the creation of an essential catalytic loop tyrosine mutant in *S. cerevisiae*. If we assume that the APRT-F74V mutation is more likely to reduce the rate of the forward reaction and potentially increase the reverse reaction, then the effect of the mutation should be to reduce the rate of AMP (and PPi) production by this route and require greater flux through the *de-novo* pathways. As these are more energy demanding, this will increase the flux through phospho-fructokinase, which is known to be controlled by the energy charge in the cell. The consequence of this would not only be an increase in the rate of glycolysis and fermentation, as observed by Zhou et al., but also a reduction in the intra-cellular concentration of phospho-sugars, which are thought to be key CCR signaling molecules (Figure 11).

Interestingly, over-expression of wild-type Aprt partially restored some CCR, even in the background of the *ptsG* and *rbsR* mutations suggesting that both EIIA^*Glu*^ and phospho-sugars are involved in CCR signaling. However, this also seemed to affect growth on xylose as a sole source of carbon, which is less easy to explain. If over-expression results in excessive production of AMP, this could have an effect on the energy charge but could also increase the flux of ribose-5-P from the pentose phosphate cycle, both of which could affect anaerobic growth on xylose in a manner which is independent of catabolite repression.

#### 4.2.5. Other mutations

The mutations highlighted above are those that can most clearly be linked to the release of CCR. Of the other common mutations, *holB* and *mutS* mutations affect DNA replication and are likely to contribute to an increased mutation rate. *Spo0A, rsfA, comEC, degU* and *degV* mutations will moderate the cell response to starvation as a consequence of using a non-metabolisable CCR analog, while the *DDX/DHX* mutation could affect regulation of a range of functions. The other common mutations affect metabolic/biosynthetic functions and are less easy to explain without further studies. *GltB* encodes a component of glutamate synthase, while *proS* encodes a proline-tRNA ligase and *dal* encodes a d-alanine racemase, but all of the mutations found were missense, so they may not have resulted in loss of function. The most intriguing in that respect is the frame-shift mutation in *hypE*, which encodes a maturation enzyme for [NiFe] hydrogenases and is clearly essential for hydrogenase activity.

## 5. Conclusion

It has generally been believed that PTS components are involved in CCR in Enterobacteriaceae, typically *via* inducer exclusion mediated by EIIA, but not in Firmicutes, whose inducer exclusion relies on the interaction of HPr-Ser-P with ABC transporters as in *B. subtilis* (Deutscher et al., 2014). However, evidence from a previous study in *B. subtilis* suggested that a functional EII^*Glu*^ is indispensable for CCR in *B. subtilis*, though the detailed mechanisms were unclear (Bachem et al., 1997). This study clearly points toward the phosphorylation state of EIIA^*Glu*^ or EIIB^*Glu*^ being important for CCR signaling in *P. thermoglucosidasius* DSM 2542 and possibly in the wider community of Firmicutes. Further study will be required to investigate the detailed mechanism. At an applied level, mutation in EIIB^*Glu*^ provides a route to release catabolite repression in this important thermophile.

## Data availability statement

The data presented in the study are deposited in the NCBI Sequence Read Archive Repository, accession number PRJNA866999.

## Author contributions

Laboratory experiments were formulated by JL, DL, and AB. Methodologies were designed by JL, DL, and RK. Experimental work was carried out by JL. The manuscript was written by JL and edited by DL, AB, and RK. All authors read and approved the final manuscript.

## Funding

This work was funded by the European Union's Horizon 2020 research and innovation programme under the Marie Skłodowska-Curie grant agreement [H2020-MSCA-CO-FUND 665992]; by Corbion and by the EPSRC [EP/L016354/1].

## Conflict of interest

Author RK was employed by Corbion. The remaining authors declare that the research was conducted in the absence of any commercial or financial relationships that could be construed as a potential conflict of interest.

## Publisher's note

All claims expressed in this article are solely those of the authors and do not necessarily represent those of their affiliated organizations, or those of the publisher, the editors and the reviewers. Any product that may be evaluated in this article, or claim that may be made by its manufacturer, is not guaranteed or endorsed by the publisher.
