# Supplementary material for: Removing carbon catabolite repression in Parageobacillus thermoglucosidasius DSM 2542
Source: Front Microbiol. 2022 Oct 20;13:985465. doi: 10.3389/fmicb.2022.985465 (PMC9631020; doi:10.3389/fmicb.2022.985465)
Supplement: Supplementary file 1 [file Data_Sheet_1.PDF]

Supplementary Material

1 PJL-*PTSH1* AND PJL-*CRH1* CHROMOSOMAL INTEGRATION PROTOCOL

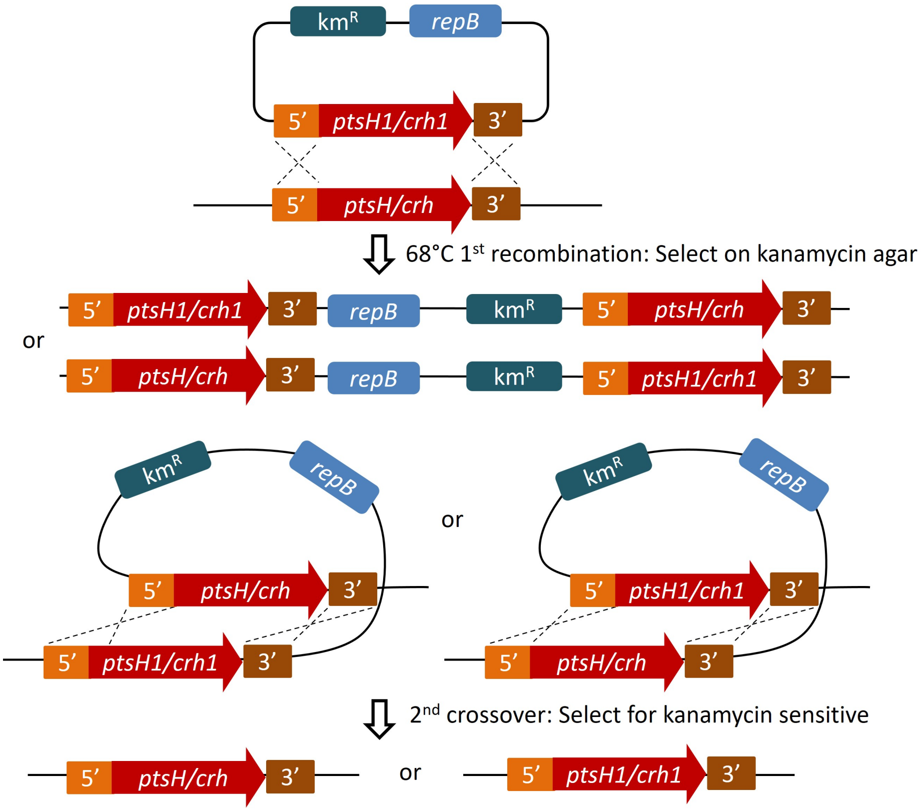

Figure S1. pJL-*ptsH1* and pJL-*crh1* chromosomal integration protocol. The *ptsH1* or *crh1* were inserted between the wild-type 5 and 3 flanking regions on pG2K-*oriT*-*bgl*-*sfgfp*-GG. The primary integration events relied on homologous recombination between either the 5' or the 3' plasmid-borne and chromosomal flanking region. The plasmid replication origin *repB* was inactive at 68°C, and therefore cells could only grow on kanamycin at 68°C when the plasmid was integrated with the chromosome. Colonies selected in the absence of kanamycin after the second crossover had either reverted to the wild type, or acquired the mutation successfully. Kanamycin sensitive colonies were identified by replica plating on plates with and without kanamycin (12.5 μg/mL). km^R: kanamycin resistance marker; *repB*: plasmid replication origin.

2 HPR-S46A AND CRH-S46A SITE-DIRECTED MUTAGENESIS IN *P. THERMOGLUCOSIDASIUS* DSM 2542

The *ptsH1* mutation removed a *Bsu15*I restriction site in *ptsH*, while the *crh1*mutation introduced an *Alu*I restriction site in *crh* (Fig.S2a). Therefore, successful site-directed mutagenesis of the *ptsH* and the *crh* genes could be confirmed by digesting the PCR products with *Bsu15*I and *Alu*I respectively. The wild type *ptsH* PCR product contained three *Bsu15*I sites. However, as one of the sites was only 25 bp away from one end and not visible on the gel, only three bands (803 bp, 478 bp and 382 bp) could be seen after *Bsu15*I digestion of the wild-type *ptsH* PCR product, while 2 bands (1281 bp and 382 bp) were seen with the

ptsH1 PCR product (Fig.S2b). Likewise, the PCR product amplified from the wild type *crh* contained one *Alu*I sites, so *Alu*I digestion of this resulted in two bands (1328 bp and 534 bp) on the gel, while the *crh1* mutation created a new *Alu*I site, producing three bands (968 bp, 534 bp and 360 bp) on the gel (Fig.S2c).

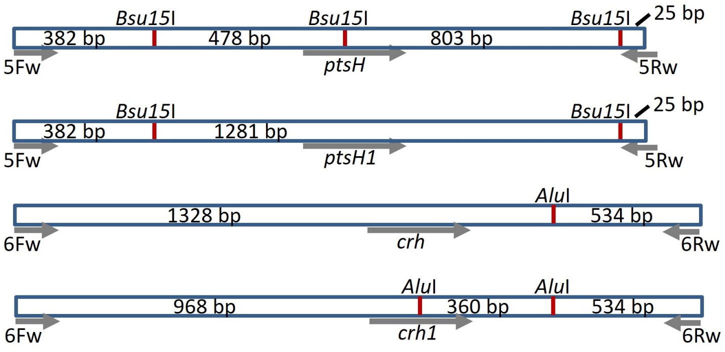

Figure S2a. Schematic diagram of the restriction sites.

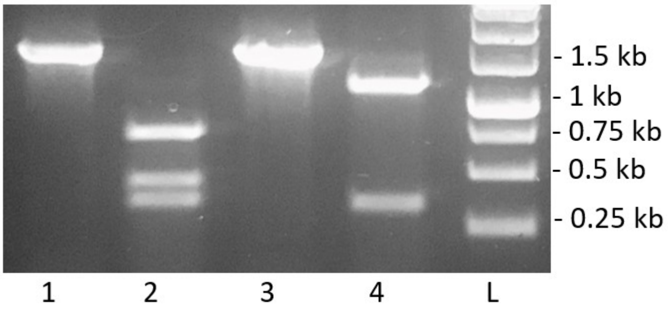

Figure S2b. *ptsH1* mutation.

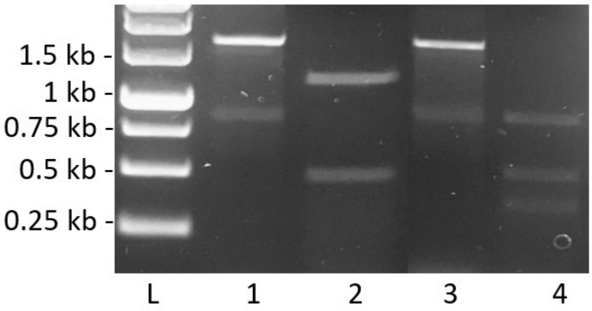

Figure S2c. *crh1* mutation.

Figure S2. *ptsH1* and *crh1* point mutation in *P. thermoglucosidasius* DSM 2542 diagnosed by PCR and enzymatic digestion shown on a 1% agarose gel. (a) Schematic diagram of the restriction sites before and after the *ptsH1* and *crh1* mutation. (b) PCR products of *ptsH1* (lane 1) and wild type *ptsH* (lane 3) with their flanking regions digested with *Bsu*15I (lane2: *ptsH1* after digestion; lane4: wild type *ptsH* after digestion). (c) PCR products of wild type *crh* (the upper band in lane 1) and *crh1* (the upper band in lane 3) with their flanking regions digested by *Alu*I (lane2: wild type *crh* after digestion; lane4: *crh1* after digestion). The lower bands in lane 1 and lane 3 were resulted by mis-priming. L: Molecular weight DNA ladder.

3 METHYLGLYOXAL AND ASM MEDIUM REACTION ASSAY

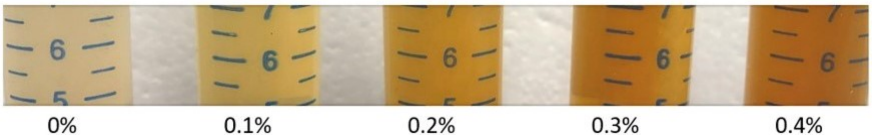

Figure S3a. *P. thermoglucosidasius* DSM 2542 culture incubated in ASM medium (1%(w/v) glucose and 1%(w/v) xylose) containing 0% , 0.1%, 0.2%, 0.3% and 0.4% methylglyoxal (v/v) respectively at 60°C after overnight incubation.

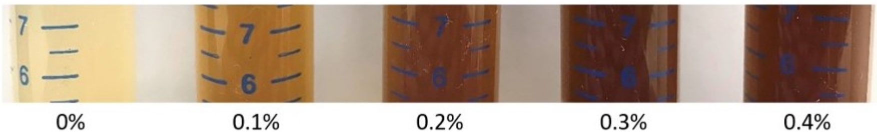

Figure S3b. *P. thermoglucosidasius* DSM 2542 culture incubated in ASM medium (1%(w/v) glucose and 1%(w/v) xylose) containing 0% , 0.1%, 0.2%, 0.3% and 0.4% methylglyoxal (v/v) respectively at 60°C overnight after 36 hours incubation.

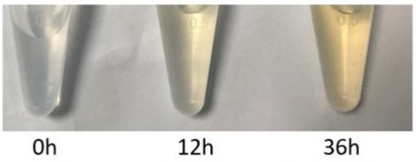

Figure S3c. Colour change of culture medium of DSM 2542 during fermentation in ASM medium (1%(w/v) glucose and 1%(w/v) xylose) without methylglyoxal.

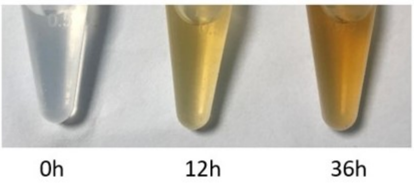

Figure S3d. Colour change of culture medium of DSM 2542 *ptsH1* during fermentation in ASM medium (1%(w/v) glucose and 1%(w/v) xylose) without methylglyoxal.

Figure S3. Methylglyoxal and ASM medium reaction assay. *P. thermoglucosidasius* DSM 2542 culture was incubated in ASM medium (1%(w/v) glucose and 1%(w/v) xylose) containing 0% , 0.1%, 0.2%, 0.3% and 0.4% methylglyoxal (v/v) respectively at 60°C. Colour change of the culture medium was recorded after (a) overnight and (b) 36 hours incubation. Panel (c) and (d) show the colour change of the culture medium of DSM 2542 and DSM 2542 *ptsH1* respectively, during fermentation in ASM medium (1%(w/v) glucose and 1%(w/v) xylose) without methylglyoxal at 60°C for 36 hours.

4 PCR AMPLIFICATION CONFIRMING THE PRESENCE OF PLASMID PJL-*PTSG*$^{+}$, PJL-*APT*$^{+}$ AND PJL-*RBSR*$^{+}$ IN *P. THERMOGLUCOSIDASIUS* 2DG-ADE2B

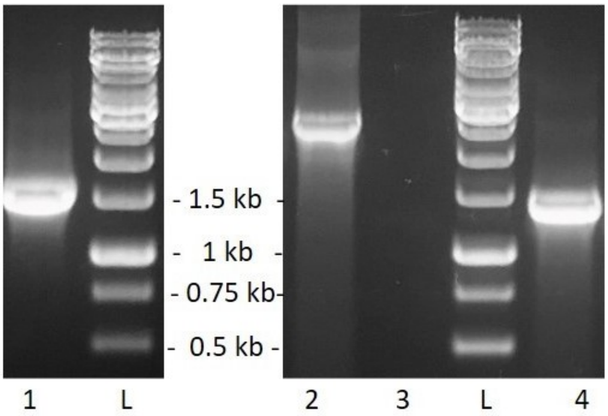

Figure S4. PCR amplification to confirming presence of plasmid pJL-*rbsR*$^{+}$, pJL-*ptsG*$^{+}$ and pJL-*apt*$^{+}$ in 2DG-ADE2b respectively. Amplicons are shown on a 1% agarose gel corresponding to successful integration of wild type *rbsR* (lane1), *ptsG* (lane2) and *apt* (lane4) from *P. thermoglucosidasius* DSM 2542 into plasmid pG2K-*oriT*-*bgl*-*sfgfp*-GG respectively. 2DG-ADE2b (lane 3) was used as the negative control. L: Molecular weight DNA ladder.

5 SINGLE NUCLEOTIDE POLYMORPHISMS (SNPS) IDENTIFIED IN CATABOLITE DE-REPRESSED MUTANT STRAINS

Table S1. SNPs in catabolite de-repressed mutant strains after continuous adaptive evolution in ASM medium with 1% xylose and 0.5% 2-DG. Tick marks indicate the presence of specific SNPs.

SNPs in CDS	Mutation Type	2DG-ADE1a	2DG-ADE1b	2DG-ADE1c	2DG-ADE1d	2DG-ADE1e
ptsP	Missense A19E	√	√	√	√	√
SsuA	Missense G138E	√				
aprt	Missense F74V	√	√	√	√	√

Table S2. SNPs in catabolite de-repressed mutant strains after adaptive evolution with intermediate selection for rapid growth on glucose. Tick marks indicate the presence of specific SNPs.

SNPs in CDS	Mutation Type		2DG-ADE2a	2DG-ADE2b	2DG-ADE2c	2DG-ADE2d	2DG-ADE2e
DDX/DHX	Missense	R100C	√	√	√	√	√
degV	Frame-shift	K208			√	√	√
degU	Start-lost	V1		√	√	√	√
rbsR	Stop-gained	T58*	√	√	√	√	√
holB	Missense	S22N	√	√	√	√	√
spoIID	Silent	R44					√
pepX	Frame-shift	PI421	√				
luxR	Frame-shift	L7	√				
PDR5	Frame-shift	V40C	√				
MFS transporter gene	Missense	L278W		√	√	√	√
ISL3 family transposase gene	Silent	A177	√				
terC	Missense	W103R	√				
ptsG	Missense	R452C	√	√	√	√	√
phoH	Missense	R312H	√				
bshC	Missense	Q474R	√				
sigE	Frame-shift	I108	√				
proS	Missense	P553S		√	√	√	√
mutS	Stop-gained	Q10*	√	√	√	√	√
gltB	Missense	E1182K	√	√	√	√	√
ureB	Missense	P3S	√				
dal4	Missense	A128V		√	√	√	√
narZ	Missense	G191D	√				
hypE	Frame-shift	G218	√	√	√		√
GGCT	Missense	T63A					√
SLC11A2	Frame-shift	N5					√
AACS	Missense	G144R			√		
dnaQ	Frame-shift	K51K					√
PDHA1	Frame-shift	I122	√				
GLA	Missense	G561D	√				
GLUD1	Missense	V413A			√		
MMUT	Missense	G443R	√				
BCKDHB	Missense	E88D	√				
spo0A	Missense	G37S	√	√	√	√	√
pgpH	Frame-shift	L23F					√
comEC	Frame-shift	N611K	√		√	√	√
rsfA	Frame-shift	K84		√	√	√	√
aprt	Missense	F74V	√	√	√	√	√
SAM1	Missense	A159T	√				
